# Static anti-windup compensator design for locally Lipschitz systems under input and output delays

**DOI:** 10.1371/journal.pone.0283734

**Published:** 2023-04-11

**Authors:** Muhammad Jazib Hameed, Muhammad Rehan, Muhammad Iqbal, Muntazir Hussain, Najam us Saqib, Jamshed Iqbal

**Affiliations:** 1 Department of Electrical Engineering, Pakistan Institute of Engineering and Applied Sciences (PIEAS), Islamabad, Pakistan; 2 Department of Computer Science, National University of Technology (NUTECH), Islamabad, Pakistan; 3 Department of Electrical and Computer Engineering, Air University, Islamabad, Pakistan; 4 School of Computer Science, Faculty of Science and Engineering, University of Hull, Hull, United Kingdom; Hebei University of Technology, CHINA

## Abstract

This paper proposes a static anti-windup compensator (AWC) design methodology for the locally Lipschitz nonlinear systems, containing time-varying interval delays in input and output of the system in the presence of actuator saturation. Static AWC design is proposed for the systems by considering a delay-range-dependent methodology to consider less conservative delay bounds. The approach has been developed by utilizing an improved Lyapunov-Krasovskii functional, locally Lipschitz nonlinearity property, delay-interval, delay derivative upper bound, local sector condition, *L*_2_ gain reduction from exogenous input to exogenous output, improved Wirtinger inequality, additive time-varying delays, and convex optimization algorithms to obtain convex conditions for AWC gain calculations. In contrast to the existing results, the present work considers both input and output delays for the AWC design (along with their combined additive effect) and deals with a more generic locally Lipschitz class of nonlinear systems. The effectiveness of the proposed methodology is demonstrated via simulations for a nonlinear DC servo motor system, possessing multiple time-delays, dynamic nonlinearity and actuator constraints.

## 1. Introduction

Input saturation nonlinearity arises when a controller produces a signal that is higher in strength than an actuator’s limits. This problem is faced by most of the practical systems because an actuator is unable to provide an unlimited power to a system. Actuator saturation causes the so-called windup phenomenon, which can result in the performance degradation of the closed-loop system. This phenomenon can cause overshoots, undershoots, time-lags, oscillations and in some cases instability [1–4] in the closed-loop response. Several incidents such as YF-22 crash, Gripen crash, and meltdown of Chernobyl power plant have been reported that occurred because of an inappropriate handling of the input saturation [4, 5]. In order to mitigate the adverse effects incurred by windup phenomenon, an anti-windup compensator (AWC) is utilized [4–6], which recovers the closed-loop system’s performance for dealing with the saturation effects. AWC design has become a heuristic research problem for the past few decades, and a lot of efforts have been accounted by researchers to evolve novel compensation techniques.

Gigantic literature is available for designing the AWC for the linear systems [5]. Robust control for the delayed nonlinear systems under actuator saturation is proposed in [7]. An approach is presented in [8] that utilizes a feedback technique to design a static AWC for ensuring global stability of the linear systems having input saturation. Recent works have considered the design of AWC for the nonlinear systems. For instance, a methodology is suggested in [9] to design the AWC for partially linearizable nonlinear systems. An AWC along with an anti-disturbance controller for the nonlinear Markovian jump systems, having multiple disturbances and input saturation nonlinearity, is designed by incorporating the *H*_∞_ control scheme and stochastic Lyapunov-Krasovskii functional (LKF) [10]. A robust nonlinear predictive control is designed for the synchronous motors having an input saturation by cascading AWC with proportional integral controller in [11]. The work of [12] includes the design of a static AWC that guarantees regional stability for the multivariable nonlinear systems by including actuator saturation via a global sector condition. Design of a static AWC, based on linear matrix inequalities (LMIs), has been presented in [13, 14] for nonlinear systems to ensure local and global stability for the closed-loop system via a generalized sector condition.

In the recent decades, time-delay has become an active area of interest for the researchers due to its presence in several natural and physical systems like chemical processes, electrical circuits, power systems, control models, nuclear reactors, communication structures, and neural networks [15–19]. Time-delays have adverse effects on a system’s performance and stability, as observed in [20]. Necessary and sufficient conditions for the delay-independent stability of linear time-invariant systems having single delay by using frequency discretization approach is presented in [21]. Stability for the continuous-time linear systems by incorporating delays, varying with time, has been established in [22] by utilizing an innovative Lyapunov functional and Jenson’s inequality to produce less conservative outcomes. In the work [23], delay-dependent stability for the nonlinear systems, containing time-varying delays with bounded derivatives, has been obtained in terms of LMIs by using improved Lyapunov functional and Wirtinger inequality.

Time-delay with input saturation has more challenging effects on a system’s behavior and stability [24]. Static AWC for linear systems with input delay is designed in the literature [25] by including global and local stability conditions for the closed-loop system in terms of LMIs. Output feedback and robust fuzzy controller for the delayed nonlinear systems with input constraints are proposed in [26]. Dynamic AWC is designed in [24] for a nonlinear system, having time-varying state delay, by using both decoupling AWC and IMC-based AWC architectures and by utilizing Lipschitz condition, Lyapunov functional and *L*_2_ gain minimization. To attain the alleviation for windup effects in nonlinear systems with time-delays, the dynamic anti-windup methodology of [24] has been extended in [27] by using Lipschitz reformulation and LPV techniques. The main problem in the dynamic windup compensation technique is that it is not realizable in the circumstances where the state of a system is not available for AWC. Recently, static anti-windup scheme is proposed in [28] for the delayed nonlinear plants by employing reformulated Lipschitz condition and Wirtinger-based inequality; however, the results are applicable to a less general class of systems. Although, many aspects of time-delay systems have been addressed in the literature [15–23]; however, specifically, nonlinear time-delay systems with input saturation have not attained much research attention in the past decade. Therefore, further struggles are obligatory to explore new control and compensation schemes for nonlinear systems with input saturation and time-delay to address the aforementioned challenges.

In this paper, we have explored a static AWC design approach for the nonlinear systems by incorporating multiple locally Lipschitz nonlinearities along with multiple time-varying interval delays at the input and output of a system. A two-step controller design methodology has been considered, where a nominal feedback controller can be employed first to achieve the required performance measures without considering actuator saturation, and then a static AWC gain can be determined in the second step to deal with the saturation nonlinearity. The results are attained in the form of nonlinear routines which can be solved via recursive linear-matrix inequalities by incorporating improved LKF, locally Lipschitz nonlinearity bounds, local sector condition, delay derivative upper bound, delay-interval, *L*_2_ gain minimization, cone complementary linearization, and improved Wirtinger based inequality. Herein, local stability conditions are derived for designing the static AWC for input and output delayed nonlinear generalized Lipschitz systems. Theoretical results are then verified by simulating the nonlinear DC motor system’s dynamics by involving nonlinearities and time-delays. Contrary to the approaches explained in [7–9, 27], a static AWC is designed for nonlinear systems with multiple time-delays. In addition, time-varying delays both in input and output of a system with different delay-ranges have been incorporated to design the static AWC as compared to existing approaches [12, 24–28]. Unlike to existing methodologies in [21–23, 29], the proposed method incorporates delay-range-dependent scheme to include both large and small delays, simultaneously. A novel technique, based on an improved Wirtinger inequality, has been applied to develop better approximation of delay terms as compared to Jenson’s inequality [22]. Further, to include broader spectrum of nonlinear functions, locally Lipschitz nonlinearity has been included in our study as opposed to the globally Lipschitz nonlinearities and conventional methods discussed in the literature [24, 27–32]. The aim of our paper is to deal with multiple time-varying delays appearing at both input and output of the nonlinear systems in the presence of actuator saturation. The prime offerings of this paper are summarized as follows:

1. To the best of our knowledge, a static AWC design scheme for a generic class of the locally Lipschitz nonlinear time-delay systems is addressed for the first time. Most of the exiting works for dealing with nonlinear systems apply simple Lipschitz condition [24, 27, 28, 31, 32], while the proposed approach can be considered for a broader scope of AWC design.
2. In comparison to existing works [12, 22, 24–28], both input and output multiple delays are considered. Most of the existing works consider either input, output or state delays, and consideration of multiple time-delays is more practical in physical systems. An advanced delay handling method by using an improved Wirtinger inequality and using delay-range-dependent method is applied. It should be noted that such a problem involves both effects of input and output delays in separate form and in combined form (namely additive time-varying delay), posing a challenging stability analysis scenario. At the same time, such a range-dependent method can be applied to both larger and smaller delays, compared to the conventional delay-dependent methods.
3. Most of the existing works like [24, 27–32] do not consider the output nonlinearities due to nonlinear sensors and nonlinear calibration equations. The present work has considered this output nonlinearity in addition to the state nonlinearity. Also a generalized model of nonlinearity has been considered, rather than the conventional global models. In contrast to conventional global models, our approach can consider polynomials via locally Lipschitz modeling of functions.
4. In contrast to the conventional higher-order and complex AWC designs in [2, 7–9, 13, 27, 30], the present work considers a static AWC design which requires less computational efforts for its implementation. The static AWC can be implemented via a proportional gain matrix for attaining stability and performance of a control system under input saturation. Besides the rigorous Lyapunov stability conditions, a method for the computation of AWC gains using tedious algebra and LMIs is provided. The proposed AWC approach can be designed by application of convex routines.

### Notation

Standard notations are employed in this study. The symbols ‖.‖_2_ and ‖.‖ are used to represent the Euclidian and *L*_2_ norms of vectors, respectively. A positive definite matrix *S* is represented by the condition *S*>0. The notation *diag*(*s*_1_,*s*_2_,⋯*s*_*n*_) describes a diagonal matrix with *n* block-diagonal entries. For a control input *u*(*t*)∈ℝ^*m*^, $u_{sat}(t)=sat(u(t))\in {\mathbb{R}}^{m}$ indicates the saturated signal *u*_*sat*_(*t*) for the saturation nonlinearity *sat*(*u*(*t*)). The notation $\bar{u}\in {\mathbb{R}}^{m}$ denotes bounds of the saturation function. *I* and 0 are used for identity and zero matrices with appropriate dimensions. *O*_*m*×*n*_ in an expression is used to denote a matrix of zero entries with *m*×*n* blocks, for which each zero block has appropriate dimensions in accordance with the expression. This notation is employed for writing compact expressions. For a matrix *S*, *S*_(*i*)_ is used to denote the *i*^th^ row.

## 2. System description

Consider a delayed nonlinear system as

$$\begin{array}{l} \dot{x}(t)=Ax(t)+f(t,x)+Bu_{sat}(t-\iota_{1}(t)),\\ y(t)=C_{1}x(t-\iota_{2}(t))+g(x(t-\iota_{2}(t)),\\ z(t)=C_{2}x(t)+D\bar{w}(t). \end{array}$$ (1)

where *x*(*t*)∈ℝ^*n*^ represents the states of the system, $u_{sat}(t-\iota_{1}(t))\in {\mathbb{R}}^{m}$ is the saturated input with an input delay *ι*_1_(*t*), and $\bar{w}\in {\mathbb{R}}^{q}$ represents the exogenous input (reference input). The signals *y*(*t*)∈ℝ^*p*^ and *z*(*t*)∈ℝ^*s*^ are the measured and exogenous outputs, respectively. The matrices *A*,*B*,*C*_1_,*C*_2_, and *D* are the constant and known. *ι*_1_(*t*) and *ι*_2_(*t*) are the unknown time-varying delays in the input and output, respectively. *f*(*t*,*x*)∈ℝ^*n*^ and $g(x(t-\iota_{2}))\in {\mathbb{R}}^{p}$ are the nonlinear functions in state and output equations, respectively. The input delay *ι*_1_(*t*) and output delay *ι*_2_(*t*) are bounded within intervals such that $0\leq h_{1}\leq \iota_{1}(t)\leq h_{2}$ and $0\leq h_{3}\leq \iota_{2}(t)\leq h_{4}$. Further, these delays validate the derivative constraints ${\dot{\iota }}_{1}(t)\leq u_{1}<1$ and ${\dot{\iota }}_{2}(t)\leq u_{2}<1$. To achieve the desired closed-loop response, the following output feedback controller without considering effects of saturation has been considered:

$$\begin{array}{l} {\dot{x}}_{cont}=A_{cont}x_{cont}+B_{cont}(\bar{w}-y),\\ u(t)=C_{cont}x_{cont}+D_{cont}(\bar{w}-y), \end{array}$$ (2)

where *x*_*cont*_(*t*)∈ℝ^*c*^ and *u*(*t*)∈ℝ^*m*^ represent the controller states and output, respectively. $A_{cont},B_{cont},C_{cont},D_{cont}$ are the controller matrices. An AWC-based control can be considered in two design steps. In the first step, a feedback controller can be designed to meet the desired requirements, and in next step, an AWC is designed and added in the feedback controller to compensate the unwanted effects of saturation. As the purpose of the present work is to consider the second design steps, the overall controller after incorporating the anti-windup takes the form

$$\begin{array}{l} {\dot{x}}_{cont}=A_{cont}x_{cont}+B_{cont}(\bar{w}-y)+E_{c}\psi (u),\\ u(t)=C_{cont}x_{cont}+D_{cont}(\bar{w}-y),\\ \psi (u)=-sat(u(t))+u(t), \end{array}$$ (3)

where *E*_*c*_ is the AWC gain to be designed for eliminating the saturation effects. The nominal controller in (2) applies the feedback of output *y* and uses the error $\bar{w}-y$ for updating the controller state equation. The control signal is computed via the second equation, given by *u*(*t*). The AWC-based proposed controller in (3) modify the controller (2) for an additional feedback of difference between saturated and unsaturated signal, given by *ψ*(*u*). This additional feedback is applied through gain *E*_*c*_ for attaining stability and performance against saturated control signal. In this study, we assume that controller (2) is available (without considering saturation), which can be designed using existing tracking control methods. The focus of our study is to compute the additional AWC gain *E*_*c*_ for achieving stability and performance against saturation. Using (1) and (3), the overall closed-loop structure by application of $\zeta (t)={\left[\begin{array}{cc} x^{T}(t) & x_{cont}^{T}(t) \end{array}\right]}^{T}$ becomes

$$\begin{array}{l} \dot{\zeta }(t)=A_{11}\zeta (t)+B_{11}\zeta (t-\iota_{1}(t))+B_{12}\zeta (t-\iota_{2}(t))+B_{13}\zeta (t-\iota_{3}(t))\\ \;+Ff_{1}(t,x)+G_{1}g_{1}(x(t-\iota_{2}(t))+G_{2}g_{1}(x(t-\iota_{3}(t))\\ \;+W_{1}\bar{w}+W_{2}\bar{w}(t-\iota_{1}(t))+Y_{1}E_{c}\psi (u)+Y_{2}\psi (u(t-\iota_{1}(t)),\\ u(t)=C_{y}\zeta (t)+D_{1}\zeta (t-\iota_{2}(t))+D_{cont}\bar{w}-D_{cont}g_{1}(x(t-\iota_{2})),\\ z(t)=C_{s}\zeta +D\bar{w}, \end{array}$$ (4)

where

$$\begin{array}{l} A_{11}=\left[\begin{array}{cc} A & 0\\ 0 & A_{cont} \end{array}\right],B_{11}=\left[\begin{array}{cc} 0 & BC_{cont}\\ 0 & 0 \end{array}\right],B_{12}=\left[\begin{array}{cc} 0 & 0\\ -B_{cont}C_{1} & 0 \end{array}\right],\\ B_{13}=\left[\begin{array}{cc} -BD_{cont}C_{1} & 0\\ 0 & 0 \end{array}\right],F=\left[\begin{array}{c} I\\ 0 \end{array}\right],G_{1}=\left[\begin{array}{c} 0\\ -B_{cont} \end{array}\right],G_{2}=\left[\begin{array}{c} -BD_{cont}\\ 0 \end{array}\right],\\ Y_{1}=\left[\begin{array}{c} 0\\ I \end{array}\right],Y_{2}=\left[\begin{array}{c} -B\\ 0 \end{array}\right],W_{1}=\left[\begin{array}{c} 0\\ B_{cont} \end{array}\right],W_{2}=\left[\begin{array}{c} BD_{cont}\\ 0 \end{array}\right],\\ C_{y}=\left[\begin{array}{cc} 0 & C_{cont} \end{array}\right],D_{1}=\left[\begin{array}{cc} -D_{cont}C_{1} & 0 \end{array}\right],C_{s}=\left[\begin{array}{cc} C_{2} & 0 \end{array}\right]. \end{array}$$ (5)

Note that *ι*_3_(*t*) is not a new delay, it is combined effect of *ι*_1_(*t*) and *ι*_2_(*t*) such that $\iota_{3}(t)=\iota_{2}(t)+\iota_{2}(t)$. It is worth mentioning that the closed-loop system (4) is complicated to deal because of three delays, namely, input delay *ι*_1_(*t*), output delay *ι*_2_(*t*) and their combined effect *ι*_3_(*t*) (additive time-varying delay). All these three delays should be dealt in accordance with their properties, the later one, namely, additive time-delay for input output effect is most difficult as it contains the effects from both input and output delays [17, 19]. The design should consider the individual constraints for time-delays *ι*_1_(*t*) and *ι*_2_(*t*) as well as the additive time-varying delay *ι*_3_(*t*). To deal with this delay, additional terms in the Lyapunov functional can be applied for the stability analysis. The closed-loop system formed by (1) and (3) has delays, which can slow the response. The conventional control mechanisms do not account for these individual and additive time-delays, therefore, there methods consider higher values of parameters like *E*_*c*_. The higher values provide a strong feedback signal, leading to overshoots, performance degradation, and instability. Combined with the saturation nonlinearity, which also limits the amplitude of signals, the effects of individual and additive time-delays can be more swear and can lead to economic losses. The purpose of our study is to ensure the desired gain *E*_*c*_ by accounting the delay-range and input saturation. The following assumption is taken on the nonlinear functions:

***Assumption 1*** [33, 34]: The nonlinear function *f*(*t*,*x*) for the region, $\kappa_{1},\kappa_{2}\in \xi^{T}{\bar{R}}^{-1}\xi \leq 1,$ $\bar{R}={\bar{R}}^{T}>0,$ and the delayed nonlinear function $g(x(t-\iota_{2}(t)))$, for the region, ${\bar{\kappa }}_{1},{\bar{\kappa }}_{2}\in \xi^{T}{\bar{R}}^{-1}\xi \leq 1,$ satisfy the following locally Lipschitz conditions:

$$\|f(t,\kappa_{1})-f(t,\kappa_{2})\|\leq \|L_{1}(\kappa_{1}-\kappa_{2})\|\forall \kappa_{1},\kappa_{2}\in {\mathbb{R}}^{n},f(t,0)=0,\forall t\geq 0,$$ (6)

$$\|g(t,{\bar{\kappa }}_{1})-g(t,{\bar{\kappa }}_{2})\|\leq \|L_{2}({\bar{\kappa }}_{1}-{\bar{\kappa }}_{2})\|\forall {\bar{\kappa }}_{1},{\bar{\kappa }}_{2}\in {\mathbb{R}}^{n},g(t,0)=0,\forall t\geq 0,$$ (7)

where the matrices *L*_1_ and *L*_2_ are constant matrices.

The conditions in (6) and (7) can be considered as upper bounds on the corresponding nonlinearities, which can be handled by application of uncertainty modeling and robust control approaches, as observed in [24, 31]. However, the present case also includes the regional constraints $\kappa_{1},\kappa_{2}\in \xi^{T}{\bar{R}}^{-1}\xi \leq 1$ and ${\bar{\kappa }}_{1},{\bar{\kappa }}_{2}\in \xi^{T}{\bar{R}}^{-1}\xi \leq 1,$ which complicates the design. For instance, the conditions (6) and (7) can be written as $\left\|f(t,x)\|\leq \|L_{1}x\right\|$ and $\left\|g(t,x)\|\leq \|L_{2}x\right\|$ by application of $\kappa_{1}={\bar{\kappa }}_{1}=x$ and $\kappa_{2}={\bar{\kappa }}_{2}=0$. However, their validity can be achieved only if $x^{T}{\bar{R}}^{-1}x\leq 1,$ which requires regional design, Lyapunov redesign and nonlinear analysis. Hence, the proposed approach differs for the existing uncertainty-based methods [24, 31] as it deals with $\left\|f(t,x)\|\leq \|L_{1}x\right\|$ and $\left\|g(t,x)\|\leq \|L_{2}x\right\|$ under the restriction of $x^{T}{\bar{R}}^{-1}x\leq 1$. The following existing results are being used for obtaining the AWC design schemes.

**Lemma 1** [35–37]: The following condition remains valid for a local region $D(\bar{u})$:

$$\psi^{T}(u)S(v-\psi (u))\geq 0,$$ (8)

$$D(\bar{u})=\{v\in {\mathbb{R}}^{m},-\bar{u}\leq u-v\leq \bar{u}\}.$$ (9)

**Lemma 2** [38, 39]: If we have a matrix *B*>0 and a function *y* in [*c*,*d*]→*R*^*n*^ is continuously differentiable in the domain [*c*,*d*]→*R*^*n*^, then

$$\int_{c}^{d}{\dot{ƛ}}^{T}(z)B\dot{ƛ}(z)dz\geq \frac{1}{d-c}(\eta_{1}{}^{T}B\eta_{1})+\frac{3}{d-c}(\eta_{2}{}^{T}B\eta_{2}),$$ (10)

$$\begin{array}{l} \eta_{1}=-ƛ(c)+ƛ(d),\\ \eta_{2}=ƛ(c)-2/(d-c)\int_{c}^{d}ƛ(z)dz+ƛ(d). \end{array}$$

**Lemma 3** [38, 39]: For a function Θ(*α*,*R*) given as

$$\Theta (\alpha ,H)=\frac{1}{\alpha }\zeta^{T}V_{1}^{T}H\zeta V_{1}+\frac{1}{1-\alpha }\zeta^{T}V_{2}^{T}H\zeta V_{2},$$

where *H*∈ℝ^*n*×*n*^, *V*_1_,*V*_2_∈ℝ^*n*×*m*^, *ζ*∈*R*^*m*^, *α*∈(0 1). If $[\begin{array}{cc} H & T\\ {}* & H \end{array}]>0$, then it follows that

$$\min\Theta (\alpha ,H)\geq {\left[\begin{array}{c} V_{1}\zeta \\ V_{2}\zeta \end{array}\right]}^{T}[\begin{array}{cc} H & T\\ {}* & H \end{array}]\left[\begin{array}{cc} V_{1}\zeta & V_{2}\zeta \end{array}\right].$$

## 3. Anti-windup design

In this section, design conditions for AWC of nonlinear locally Lipschitz systems with multiple delays and saturation restriction have been derived. Let us define

$$\eta (t)={\left[\begin{array}{cc} z^{T}(t) & z^{T}(t-\iota_{1}) \end{array}\right]}^{T},\omega (t)={\left[\begin{array}{cc} {\bar{w}}^{T}(t) & {\bar{w}}^{T}(t-\iota_{1}) \end{array}\right]}^{T}.$$

The following theorem provides a stability analysis result for given AWC gain, which will be applied to obtain the main AWC design condition.

**Theorem 1:** Consider the nonlinear system (1) with input and output time-delays, satisfying Assumption 1. Assume that there exist a diagonal symmetric matrix *W*>0, symmetric matrices *P*>0, *Q*_*i*_>0 for (*i* = 1,…,4,7,…14) and *Z*_*j*_>0 for (*j* = 1,2,3,⋯,8) and matrices *N* and *E*_*c*_ of appropriate dimensions such that

$$\begin{array}{l} \phi^{T}{ϒ}^{1}\phi +{\dot{\zeta }}^{T}(t)[h_{2}Z_{1}+h_{2}Z_{7}+h_{12}Z_{2}+h_{12}Z_{8}+h_{4}Z_{3}+h_{4}Z_{5}+h_{34}Z_{4}+h_{34}Z_{6}]\dot{\zeta }(t)\\ -\frac{1}{h_{2}}\phi^{T}T_{1}{}^{T}\psi_{1}T_{1}\phi -\frac{1}{h_{12}}\phi^{T}T_{2}{}^{T}\psi_{2}T_{2}\phi -\frac{1}{h_{4}}\phi^{T}T_{3}{}^{T}\psi_{3}T_{3}\phi -\frac{1}{h_{34}}\phi^{T}T_{4}{}^{T}\psi_{4}T_{4}\phi \\ -\frac{1}{h_{4}}\phi^{T}T_{5}{}^{T}\psi_{5}T_{5}\phi -\frac{1}{h_{34}}\phi^{T}T_{6}{}^{T}\psi_{6}T_{6}\phi -\frac{1}{h_{2}}\phi^{T}T_{7}{}^{T}\psi_{7}T_{7}\phi -\frac{1}{h_{12}}\phi^{T}T_{8}{}^{T}\psi_{8}T_{8}\phi <0, \end{array}$$ (11)

$${ϒ}^{1}=[\begin{array}{cc} {ϒ}^{11} & {ϒ}^{12}\\ {}* & {ϒ}^{22} \end{array}],$$ (12)

$$[\begin{array}{cc} P & I\\ {}* & \hbar \bar{R} \end{array}]\geq 0.$$ (13)

$$[\begin{array}{cc} P & {\left(C_{y}{}_{\left(i\right)}-N_{\left(i\right)}\right)}^{T}\\ {}* & \hbar {\bar{u}}_{\left(i\right)}^{-1} \end{array}]\geq 0,$$ (14)

where the remaining details on various matrices can be seen in Appendix A in S1 Appendix. Then the state of the closed-loop system (4) will be asymptotically stable for all initial conditions within the elliptical region $\zeta^{T}(0)P\zeta (0)\leq 1$. Further, the *L*_2_ gain from *ω*(*t*) to *η*(*t*) will remain bounded by $\gamma =\sqrt{\rho },$ if $\|\omega (t){\|}_{2}^{2}\leq \kappa^{2}$.

***Proof*:** Consider an LKF for *t*_1_ = *t*−*t*_1_ and *t*_2_ = *t*−*t*_2_ as

$$\begin{array}{l} V(t,\zeta )=\zeta^{T}P\zeta +\int_{t-\iota_{1}}^{t}\zeta^{T}Q_{7}\zeta d\delta +\sum_{j=1}^{2}\int_{t-h_{j}}^{t}\zeta^{T}Q_{j}\zeta d\delta +\int_{-h_{2}}^{0}\int_{t+s}^{t}{\dot{\zeta }}^{T}Z_{1}\dot{\zeta }dsd\delta +\int_{-h_{2}}^{-h_{1}}\int_{t+s}^{t}{\dot{\zeta }}^{T}Z_{2}\dot{\zeta }d\delta \\ \;+\int_{t-\iota_{2}}^{t}\zeta^{T}Q_{8}\zeta d\delta +\sum_{j=3}^{4}\int_{t-h_{j}}^{t}\zeta^{T}Q_{j}\zeta d\delta +\int_{-h_{4}}^{0}\int_{t+s}^{t}{\dot{\zeta }}^{T}Z_{3}\dot{\zeta }dsd\delta +\int_{-h_{4}}^{-h_{3}}\int_{t+s}^{t}{\dot{\zeta }}^{T}Z_{4}\dot{\zeta }d\delta \\ \;+\int_{t_{1}-\iota_{2}}^{-t_{1}}\zeta^{T}Q_{9}\zeta d\delta +\int_{t_{2}-\iota_{1}}^{-t_{2}}\zeta^{T}Q_{10}\zeta d\delta +\int_{t_{1}-h_{3}}^{-t_{1}}\zeta^{T}Q_{11}\zeta d\delta +\int_{t_{1}-h_{4}}^{-t_{1}}\zeta^{T}Q_{12}\zeta d\delta +\int_{t_{2}-h_{1}}^{-t_{2}}\zeta^{T}Q_{13}\zeta d\delta \\ \;+\int_{t_{2}-h_{2}}^{-t_{2}}\zeta^{T}Q_{14}\zeta d\delta +\int_{-h_{4}}^{0}\int_{t_{1}+s}^{t_{1}}{\dot{\zeta }}^{T}Z_{5}\dot{\zeta }dsd\delta +\int_{-h_{4}}^{-h_{3}}\int_{t_{1}+s}^{t_{1}}{\dot{\zeta }}^{T}Z_{6}\dot{\zeta }dsd\delta +\int_{-h_{2}}^{0}\int_{t_{2}+s}^{t_{2}}{\dot{\zeta }}^{T}Z_{7}\dot{\zeta }dsd\delta \\ \;+\int_{-h_{2}}^{-h_{1}}\int_{t_{2}+s}^{t_{2}}{\dot{\zeta }}^{T}Z_{8}\dot{\zeta }dsd\delta \end{array}$$ (15)

Note that the first nine terms in the LKF are needed for considering the individual time-delays. The remaining ten terms have been incorporated in the proposed LKF to deal with the combined effect of time-delay (namely additive time-varying delay), which causes complexity in the stability analysis. The upper bound on the time derivative of *V*(*t*,*ζ*) by substituting (4) and delay bounds is obtained as follows:

$$\begin{array}{l} \dot{V}(t,\zeta )\leq 2\zeta^{T}(t)PA_{11}\zeta (t)+2\zeta^{T}(t)PB_{11}\zeta (t-\iota_{1})+2\zeta^{T}(t)PB_{12}\zeta (t-\iota_{2})+2\zeta^{T}(t)PB_{13}\zeta (t-\iota_{3})\\ \;+2\zeta^{T}(t)PFf(t,x)+2\zeta^{T}(t)PG_{1}g(x(t-\iota_{2}))+2\zeta^{T}(t)PG_{2}g(x(t-\iota_{3}))+2\zeta^{T}(t)PW_{1}\bar{w}(t)\\ \;+2\zeta^{T}(t)PW_{2}\bar{w}(t-\iota_{1}(t))+2\zeta^{T}(t)PY_{1}E_{c}\psi (u(t))+2\zeta^{T}(t)PY_{2}\psi (u(t-\iota_{1}(t))\\ \;+\zeta^{T}(t)Q_{7}\zeta (t)-(1-u_{1})\zeta^{T}(t-\iota_{1})Q_{7}\zeta (t-\iota_{1})+\sum_{j=1}^{2}\left\{\zeta^{T}(t)Q_{j}\zeta (t)-\zeta^{T}(t-h_{j})Q_{j}\zeta (t-h_{j})\right\}\\ \;+{\dot{\zeta }}^{T}\{h_{2}Z_{1}+h_{12}Z_{2}\}\dot{\zeta }+\zeta^{T}(t)Q_{8}\zeta (t)-(1-u_{2})\zeta^{T}(t-\iota_{2})Q_{8}\zeta (t-\iota_{2})\\ \;+\sum_{j=3}^{4}\left\{\zeta^{T}(t)Q_{j}\zeta (t)-\zeta^{T}(t-h_{j})Q_{j}\zeta (t-h_{j})\right\}+{\dot{\zeta }}^{T}\{h_{4}Z_{3}+h_{34}Z_{4}\}\dot{\zeta }\\ \;+\zeta^{T}(t-\iota_{1})Q_{9}\zeta (t-\iota_{1})-(1-u_{1}-u_{2})\zeta^{T}(t-\iota_{1}-\iota_{2})Q_{9}\zeta (t-\iota_{1}-\iota_{2})\\ \;+\zeta^{T}(t-\iota_{2})Q_{10}\zeta (t-\iota_{2})-(1-u_{1}-u_{2})\zeta^{T}(t-\iota_{1}-\iota_{2})Q_{10}\zeta (t-\iota_{1}-\iota_{2})\\ \;+\zeta^{T}(t-\iota_{1})Q_{11}\zeta (t-\iota_{1})-(1-u_{1})\zeta^{T}(t-\iota_{1}-h_{3})Q_{11}\zeta (t-\iota_{1}-h_{3})\\ \;+\zeta^{T}(t-\iota_{1})Q_{12}\zeta (t-\iota_{1})-(1-u_{1})\zeta^{T}(t-\iota_{1}-h_{4})Q_{12}\zeta (t-\iota_{1}-h_{4})\\ \;+\zeta^{T}(t-\iota_{2})Q_{13}\zeta (t-\iota_{2})-(1-u_{2})\zeta^{T}(t-\iota_{2}-h_{1})Q_{13}\zeta (t-\iota_{2}-h_{1})\\ \;+\zeta^{T}(t-\iota_{2})Q_{14}\zeta (t-\iota_{2})-(1-u_{2})\zeta^{T}(t-\iota_{2}-h_{2})Q_{14}\zeta (t-\iota_{2}-h_{2})\\ \;+{\dot{\zeta }}^{T}\{h_{4}Z_{5}+h_{34}Z_{6}+h_{2}Z_{7}+h_{21}Z_{8}\}\dot{\zeta }-\int_{t-h_{2}}^{t}{\dot{\zeta }}^{T}Z_{1}\dot{\zeta }ds-\int_{t-h_{2}}^{t-h_{1}}{\dot{\zeta }}^{T}Z_{2}\dot{\zeta }ds-\int_{t-h_{4}}^{t}{\dot{\zeta }}^{T}Z_{3}\dot{\zeta }ds\\ \;-\int_{t-h_{4}}^{t-h_{3}}{\dot{\zeta }}^{T}Z_{4}\dot{\zeta }ds-\int_{t_{1}-h_{4}}^{t_{1}}{\dot{\zeta }}^{T}Z_{5}\dot{\zeta }ds-\int_{t_{1}-h_{4}}^{t_{1}-h_{3}}{\dot{\zeta }}^{T}Z_{6}\dot{\zeta }ds-\int_{t_{2}-h_{2}}^{t_{2}}{\dot{\zeta }}^{T}Z_{7}\dot{\zeta }ds-\int_{t_{2}-h_{2}}^{t_{2}-h_{1}}{\dot{\zeta }}^{T}Z_{8}\dot{\zeta }ds. \end{array}$$ (16)

By using Lemmas 2–3 and expression in Appendix A in S1 Appendix, we can write that

$$-\int_{t-h_{2}}^{t}{\dot{\zeta }}^{T}Z_{1}\dot{\zeta }ds\leq \frac{-1}{h_{2}}\phi^{T}T_{1}^{T}\psi_{1}T_{1}\phi .$$ (17)

Similarly, the upper bound of the remaining integral terms in (16) can be approximated as

$$-\int_{t-h_{2}}^{t-h_{1}}{\dot{\zeta }}^{T}Z_{2}\dot{\zeta }ds\leq \frac{-1}{h_{21}}\phi^{T}T_{2}^{T}\psi_{2}T_{2}\phi ,$$ (18)

$$-\int_{t-h_{4}}^{t}{\dot{\zeta }}^{T}Z_{3}\dot{\zeta }ds\leq \frac{-1}{h_{4}}\phi^{T}T_{3}^{T}\psi_{3}T_{3}\phi ,$$ (19)

$$-\int_{t-h_{4}}^{t-h_{3}}{\dot{\zeta }}^{T}Z_{4}\dot{\zeta }ds\leq \frac{-1}{h_{34}}\phi^{T}T_{4}^{T}\psi_{4}T_{4}\phi ,$$ (20)

$$-\int_{t_{1}-h_{4}}^{t_{1}}{\dot{\zeta }}^{T}Z_{5}\dot{\zeta }ds\leq \frac{-1}{h_{4}}\phi^{T}T_{5}^{T}\psi_{5}T_{5}\phi ,$$ (21)

$$-\int_{t_{1}-h_{4}}^{t_{1}-h_{3}}{\dot{\zeta }}^{T}Z_{6}\dot{\zeta }ds\leq \frac{-1}{h_{34}}\phi^{T}T_{6}^{T}\psi_{6}T_{6}\phi ,$$ (22)

$$-\int_{t_{2}-h_{2}}^{t_{2}}{\dot{\zeta }}^{T}Z_{7}\dot{\zeta }ds\leq \frac{-1}{h_{2}}\phi^{T}T_{7}^{T}\psi_{7}T_{7}\phi ,$$ (23)

$$-\int_{t_{2}-h_{2}}^{t_{2}-h_{1}}{\dot{\zeta }}^{T}Z_{8}\dot{\zeta }ds\leq \frac{-1}{h_{21}}\phi^{T}T_{8}^{T}\psi_{8}T_{8}\phi .$$ (24)

By employing the inequalities (17)–(24), the upper bound on $\dot{V}(t,\zeta )$ via the condition (16) can be written as

$$\begin{array}{l} \dot{V}(t,\zeta )\leq 2\zeta^{T}(t)PA_{11}\zeta (t)+2\zeta^{T}(t)PB_{11}\zeta (t-\iota_{1})+2\zeta^{T}(t)PB_{12}\zeta (t-\iota_{2})+2\zeta^{T}(t)PB_{13}\zeta (t-\iota_{3})\\ \;+2\zeta^{T}(t)PFf(t,x)+2\zeta^{T}(t)PG_{1}g(x(t-\iota_{2}))+2\zeta^{T}(t)PG_{2}g(x(t-\iota_{3}))+2\zeta^{T}(t)PW_{1}\bar{w}(t)\\ \;+2\zeta^{T}(t)PW_{2}\bar{w}(t-\iota_{1}(t))+2\zeta^{T}(t)PY_{1}E_{c}\psi (u(t))+2\zeta^{T}(t)PY_{2}\psi (u(t-\iota_{1}(t))\\ \;+\zeta^{T}(t)Q_{7}\zeta (t)-(1-u_{1})\zeta^{T}(t-\iota_{1})Q_{7}\zeta (t-\iota_{1})+\sum_{j=1}^{2}\left\{\zeta^{T}(t)Q_{j}\zeta (t)-\zeta^{T}(t-h_{j})Q_{j}\zeta (t-h_{j})\right\}\\ \;+\zeta^{T}(t)Q_{8}\zeta (t)-(1-u_{2})\zeta^{T}(t-\iota_{2})Q_{8}\zeta (t-\iota_{2})+\sum_{j=3}^{4}\left\{\zeta^{T}(t)Q_{j}\zeta (t)-\zeta^{T}(t-h_{j})Q_{j}\zeta (t-h_{j})\right\}\\ \;+\zeta^{T}(t-\iota_{1})Q_{9}\zeta (t-\iota_{1})-(1-u_{1}-u_{2})\zeta^{T}(t-\iota_{1}-\iota_{2})Q_{9}\zeta (t-\iota_{1}-\iota_{2})\\ \;+\zeta^{T}(t-\iota_{2})Q_{10}\zeta (t-\iota_{2})-(1-u_{1}-u_{2})\zeta^{T}(t-\iota_{1}-\iota_{2})Q_{10}\zeta (t-\iota_{1}-\iota_{2})\\ \;+\zeta^{T}(t-\iota_{1})Q_{11}\zeta (t-\iota_{1})-(1-u_{1})\zeta^{T}(t-\iota_{1}-h_{3})Q_{11}\zeta (t-\iota_{1}-h_{3})\\ \;+\zeta^{T}(t-\iota_{1})Q_{12}\zeta (t-\iota_{1})-(1-u_{1})\zeta^{T}(t-\iota_{1}-h_{4})Q_{12}\zeta (t-\iota_{1}-h_{4})\\ \;+\zeta^{T}(t-\iota_{2})Q_{13}\zeta (t-\iota_{2})-(1-u_{2})\zeta^{T}(t-\iota_{2}-h_{1})Q_{13}\zeta (t-\iota_{2}-h_{1})\\ \;+\zeta^{T}(t-\iota_{2})Q_{14}\zeta (t-\iota_{2})-(1-u_{2})\zeta^{T}(t-\iota_{2}-h_{2})Q_{14}\zeta (t-\iota_{2}-h_{2})\\ \;+{\dot{\zeta }}^{T}(t)\{h_{2}Z_{1}+h_{12}Z_{2}+h_{4}Z_{3}+h_{34}Z_{4}+h_{4}Z_{5}+h_{34}Z_{6}+h_{2}Z_{7}+h_{21}Z_{8}\}\dot{\zeta }(t)\\ \;-\frac{1}{h_{2}}\phi^{T}T_{1}{}^{T}\psi_{1}T_{1}\phi -\frac{1}{h_{4}}\phi^{T}T_{3}{}^{T}\psi_{3}T_{3}\phi -\frac{1}{h_{34}}\phi^{T}T_{4}{}^{T}\psi_{4}T_{4}\phi -\frac{1}{h_{4}}\phi^{T}T_{5}{}^{T}\psi_{5}T_{5}\phi \\ \;-\frac{1}{h_{34}}\phi^{T}T_{6}{}^{T}\psi_{6}T_{6}\phi -\frac{1}{h_{2}}\phi^{T}T_{7}{}^{T}\psi_{7}T_{7}\phi -\frac{1}{h_{12}}\phi^{T}T_{2}{}^{T}\psi_{2}T_{2}\phi -\frac{1}{h_{12}}\phi^{T}T_{8}{}^{T}\psi_{8}T_{8}\phi . \end{array}$$ (25)

To ensure the *L*_2_ gain minimization from an exogenous input $\bar{w}(t)$ to an output *z*(*t*), we consider the following objective inequality:

$$\begin{array}{l} J_{1}(t,\zeta )=\dot{V}+z^{T}z+z^{T}(t-\iota_{1})z(t-\iota_{1})\\ \;-\gamma^{2}{\bar{w}}^{T}\bar{w}-\gamma^{2}{\bar{w}}^{T}(t-\iota_{1})\bar{w}(t-\iota_{1})\\ \;<0. \end{array}$$ (26)

Using (6)–(9), and (26), it reveals that

$$\begin{array}{l} J_{2}(t,\zeta )=J_{1}(t,\zeta )+\psi^{T}(u)W(v-\psi (u))+{\left(v-\psi (u)\right)}^{T}W\psi (u)\\ \;+\psi^{T}u(t-\iota_{1})W(v(t-\iota_{1})-\psi (t-\iota_{1}))\\ \;+{\left(v(t-\iota_{1})-\psi (t-\iota_{1})\right)}^{T}W\psi (u(t-\iota_{1}))\\ \;-f^{T}(t,x)f(t,x)+\zeta^{T}L_{1}^{T}L_{1}\zeta \\ \;-g^{T}(x(t-\iota_{2}))g(x(t-\iota_{2}))+\zeta^{T}(t-\iota_{2})L_{2}^{T}L_{2}\zeta (t-\iota_{2})\\ \;-g^{T}(x(t-\iota_{3}))g(x(t-\iota_{3}))+\zeta^{T}(t-\iota_{3})L_{2}^{T}L_{2}\zeta (t-\iota_{3})\\ \;<0. \end{array}$$ (27)

If *J*_2_(*t*,*ζ*)<0, then *J*_1_(*t*,*ζ*)<0 is guaranteed under Assumption 1 and Lemma 1. As the region specified by Lemma 1 shows that it is strictly positive so we can say that the regional analysis presented by *J*_1_(*t*,*ζ*)<0 is valid for *J*_2_(*t*,*ζ*)<0. Further by using (4), (25), and (27) and choosing the auxiliary vector as $v=N\zeta (t)+D_{1}\zeta (t-\iota_{2}(t))+D_{cont}\bar{w}-D_{cont}g(x(t-\iota_{2}))$, one can obtain (11). Furthermore, the following two cases can be deduced from expressions (26) and (27).

**Case I:** If $\bar{w}=0$, then from (26), one obtains that $\dot{V}(t,\zeta )<0$ for every initial condition in $\zeta^{T}(0)P\zeta (0)\leq 1$. It ensures that the closed-loop system’s states remain in the region $\zeta^{T}(t)P\zeta (t)\leq 1$ and will converge to the origin.

**Case II:** If initial condition is zero, that is, *ζ*(0) = 0, then (26)–(27) ensures that the *L*_2_ gain from *ω*(*t*) to *η*(*t*) is always less than *γ* for $\|\omega (t){\|}_{2}^{}\leq \kappa$ because *V*(*t*,*ζ*)>0. This result can be obtained by integrating (26) and using

$$\eta (t)={\left[\begin{array}{cc} z^{T}(t) & z^{T}(t-\iota_{1}) \end{array}\right]}^{T},\omega (t)={\left[\begin{array}{cc} {\bar{w}}^{T}(t) & {\bar{w}}^{T}(t-\iota_{1}) \end{array}\right]}^{T}.$$

Further, integrating *J*_1_(*t*,*ζ*)<0, we have $\zeta^{T}(t)P\zeta (t)\leq \gamma^{2}\kappa^{2}.$ Choosing $\gamma^{2}\kappa^{2}=\hbar^{-1}$, we attain the region where the states of the closed-loop system remain bounded for all time such that $\zeta^{T}(t)\hbar P\zeta (t)\leq 1$. Including the region of convergence $\zeta^{T}(t)\hbar P\zeta (t)\leq 1$ into $\zeta^{T}(t){\bar{R}}^{-1}\zeta (t)\leq 1$ by using the inequality $\hbar P\geq {\bar{R}}^{-1}$ and, further, applying the Schur complement to $P-\hbar^{-1}{\bar{R}}^{-1}\geq 0$, we achieve the condition in (13). For $v=N\zeta (t)+D_{1}\zeta (t-\iota_{2}(t))+D_{cont}\bar{w}-D_{cont}g(x(t-\iota_{2}))$, the local region in Lemma 1 can be written as $D(\bar{u})=\{\zeta \in {\mathbb{R}}^{n+c},||(C_{y}{}_{\left(i\right)}-N_{\left(i\right)})\zeta |\leq {\bar{u}}_{\left(i\right)}\}.$ Equivalently, we have ${\bar{u}}_{\left(i\right)}^{-1}\zeta^{T}{\left(C_{y}{}_{\left(i\right)}-N_{\left(i\right)}\right)}^{T}(C_{y}{}_{\left(i\right)}-N_{\left(i\right)})\zeta \leq 1$. By including the region $\zeta^{T}(t)\hbar P\zeta (t)\leq 1$ into ${\bar{u}}_{\left(i\right)}^{-1}\zeta^{T}{\left(C_{y}{}_{\left(i\right)}-N_{\left(i\right)}\right)}^{T}(C_{y}{}_{\left(i\right)}-N_{\left(i\right)})\zeta \leq 1$, we obtain the relation

$$\hbar P-{\bar{u}}_{\left(i\right)}^{-1}{\left(C_{y}{}_{\left(i\right)}-N_{\left(i\right)}\right)}^{T}(C_{y}{}_{\left(i\right)}-N_{\left(i\right)})\geq 0.$$ (28)

Furthermore, by applying the Schur complement, we reach at (14).

**Remark 1:** Looking at the literature, numerous dynamic AWC methodologies, for instance, [2, 7–9, 13, 27, 30, 37], have been suggested to alleviate the saturation effects in nonlinear systems with or without delay. Besides the dynamic AWC, we have proposed a static AWC for the nonlinear plants with input and output delay. The proposed static AWC is attractive owing to numerous features: Firstly, the dynamic AWC methodologies consider the assumption that all states of a system are available to the compensator block, however, this assumption may not be fulfilled in some practical applications. The anticipated static AWC does not require the states of a system for the compensation purpose. Secondly, in contrast to the dynamic AWC, the static AWC is computationally uncomplicated. Thirdly, the implementation of the static AWC is comparatively very easy.

**Remark 2:** The design of an AWC for the nonlinear globally Lipschitz systems is considered in the literature [24, 27, 28, 31, 37]. However, it is important to note that mostly nonlinear systems do not validate the so-called global Lipschitz condition. For instance, the nonlinear functions *f*(*t*,*x*) = −*x*^2^ and *f*(*t*,*x*) = −*x*^3^ are locally Lipschitz on ℝ, but not globally Lipschitz because $\dot{f}(t,x)=-2x$ and $\dot{f}(t,x)=3x$ are not globally bounded. In this research work, the design of a static AWC for the locally Lipschitz delayed nonlinear systems has been addressed for the first time. The local AWC design is less conservative and is applicable to a broader form of systems as compared to the global AWC results. In addition, the presented work considers nonlinearities both at the state and at the output equations in contrast to existing works.

Now, we provide the proposed AWC design framework in the following theorem:

**Theorem 2.** Consider the nonlinear system (1) with input and output time-delays, satisfying Assumption 1. Assume that there exist a diagonal symmetric matrix *U*>0, symmetric matrices *X*>0, ${\tilde{Q}}_{i}>0$ for (*i* = 1,…,4,7,…14) and ${\tilde{Z}}_{j}>0$ for (*j* = 1,2,3,⋯,8) and matrices $\bar{N}$ and *V* of appropriate dimensions such that

$$\begin{array}{l} \Delta^{2}-\frac{1}{h_{2}}T_{11}{}^{T}\psi_{1}T_{11}-\frac{1}{h_{12}}T_{22}{}^{T}\psi_{2}T_{22}-\frac{1}{h_{4}}T_{33}{}^{T}\psi_{3}T_{33}-\frac{1}{h_{34}}T_{44}{}^{T}\psi_{4}T_{44}\\ -\frac{1}{h_{4}}T_{55}{}^{T}\psi_{5}T_{55}-\frac{1}{h_{34}}T_{66}{}^{T}\psi_{6}T_{66}-\frac{1}{h_{2}}T_{77}{}^{T}\psi_{7}T_{77}-\frac{1}{h_{12}}T_{88}{}^{T}\psi_{8}T_{88}<0, \end{array}$$ (29)

$$\Delta^{2}=[\begin{array}{ccc} \Delta^{11} & \Delta^{12} & \Delta^{13}\\ {}* & \Delta^{22} & \Delta^{23}\\ {}* & * & \Delta^{33} \end{array}],$$ (30)

$$[\begin{array}{cc} X & X\\ {}* & \hbar \bar{R} \end{array}]\geq 0,$$ (31)

$$[\begin{array}{cc} X & XC_{y(t)}^{T}-{\bar{N}}_{\left(i\right)}^{T}{)}^{T}\\ {}* & \hbar {\bar{u}}_{\left(i\right)}^{-1} \end{array}]\geq 0,$$ (32)

where the selection of various matrices can be attained as in Appendix B in S1 Appendix. Then the state of the closed-loop system (4) will be asymptotically stable for all initial conditions within the elliptical region $\zeta^{T}(0)P\zeta (0)\leq 1$. The *L*_2_ gain from *ω*(*t*) to *η*(*t*) will remain bounded by $\gamma =\sqrt{\rho }$, if $\|\omega (t){\|}_{2}^{2}\leq \kappa^{2}$. The anti-windup gain can be found out using *E*_*c*_ = *VU*^−1^.

**Proof:** Applying (4), eliminating the time-dependent quantity *ϕ*(*t*) in (11), applying the congruence transformation by post- and pre-multiplying the resultant by *diag*(℧_1_,℧_2_), where ${℧}_{1}=diag(P^{-1},P^{-1},P^{-1},P^{-1},P^{-1},P^{-1},P^{-1},P^{-1},P^{-1},P^{-1},P^{-1},P^{-1}I,I,I,I,I,W^{-1},W^{-1})$ and ${℧}_{2}=(I,I,I,I,I,I,I,I,I,I,I,I)$, then substituting $P^{-1}=X,W^{-1}=U,{\tilde{Z}}_{i}=P^{-1}Z_{i}P^{-1},$ ${\tilde{Q}}_{i}=P^{-1}Q_{i}P^{-1},$ and *V* = *E*_*C*_*U*, and further applying the Schur complement, the inequality (29) is obtained. Furthermore, the conditions (31) and (32) are obtain by applying the congruence transformation with *diag*(*P*^−1^,*I*) to (13) and (14), respectively, and taking $X=P^{-1},XN^{T}=\bar{N},$ which complete the proof.

**Remark 3:** A static AWC has been designed for the linear and nonlinear systems [7–14] without considering time-delays in the dynamics. The approaches such as [24–28] have incorporated only a single time-varying delay in the system’s dynamics for designing an AWC. In contrast, this work considers multiple time-varying range-dependent delays both at input and output of the system, which also incorporates the additive time-varying delay effect in terms of $\iota_{3}(t)=\iota_{2}(t)+\iota_{2}(t)$. Such a delay-range-dependent method by considering additive time-delay can help us to deal simultaneously with all ranges of delays and multiple overlapping delays in the dynamics.

**Remark 4:** In order to approximate the delay derivatives, several mathematical inequalities like Jensen’s and Wirtinger have been used extensively in the literature [13–17, 38–40]. Herein, state of the art methods like Wirtinger inequality, additive time-delay, and convex optimization algorithms are used to obtain inequality-based conditions for the AWC gain calculations. Wirtinger inequality being mathematically complex provides a better approximation of the derivate as compared to Jensen’s inequality. We have incorporated an improved version of Wirtinger inequality in our design to achieve the less conservative results than Wirtinger inequality already being utilized in existing literature.

**Remark 5:** The proposed static AWC design condition in Theorem 2 is nonlinear in nature due to terms like $X{\tilde{Z}}_{i}^{-1}X$ for *i* = 1,2,…,8. Such terms can be handled by using cone complementary linearization approach. This approach will lead to a nonlinear optimization problem with recursive convex constraints, the optimization of which can be attained via linearization. The corresponding approach can found in the works [27, 28].

The present study has considered the AWC design for nonlinear systems with time-varying delays, validating *i*_1_(*t*)≤*u*_1_<1 and *i*_2_(*t*)≤*u*_2_<1 by application of delay-rate-dependent approach. The limitation of this approach is consideration of delay-rate less than unity. To tackle this issue, the present approach can be easily extended to the delay-rate-independent approach as in [41, 42]. Further, a better option can be consideration of delay-rate-dependent method with less conservative delay-rate restriction, as observed in [43]. In future, the present study can be extended to deal with the delay-rate restriction.

## 4. Simulation and results

**Fig 1:**
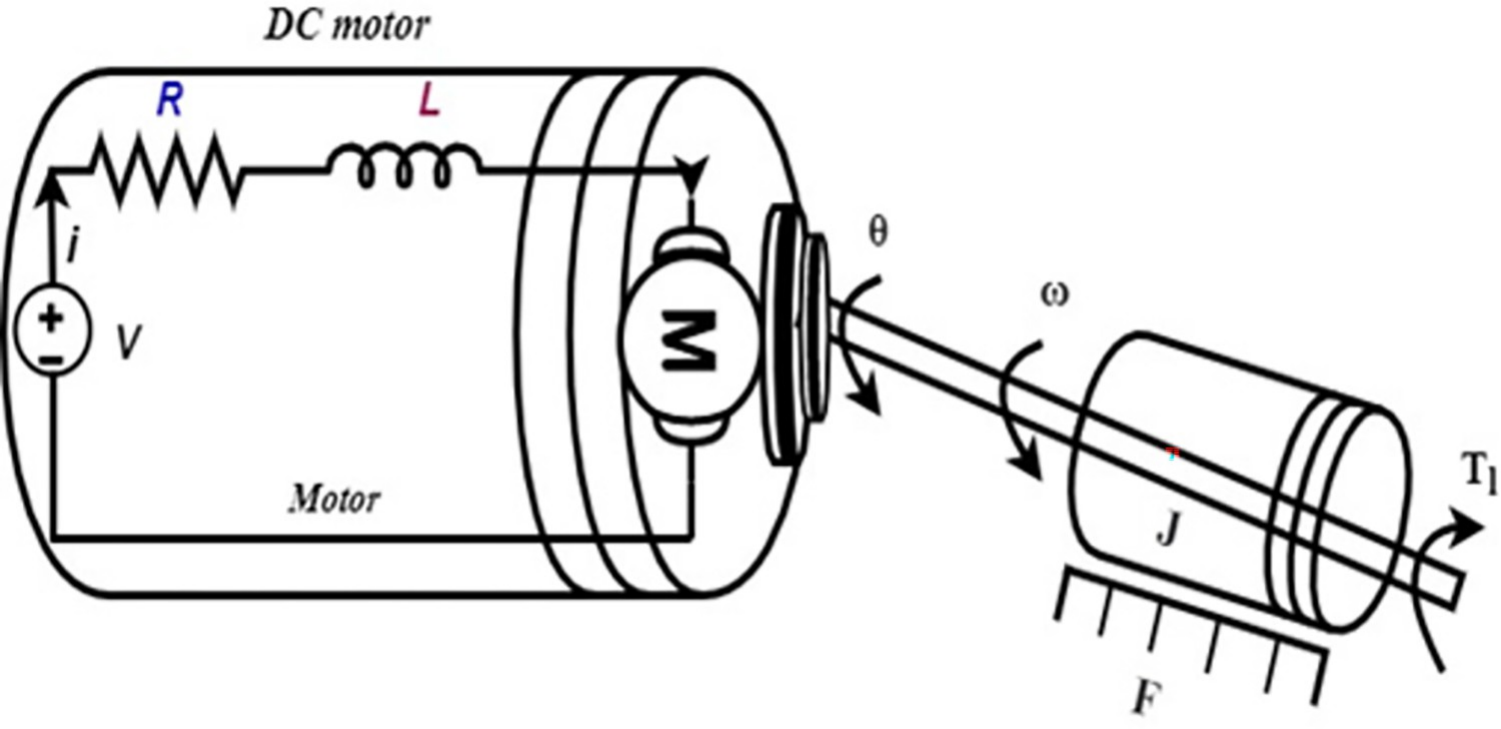
Permanent magnet DC motor.

Herein, we apply the proposed static AWC results to the nonlinear permanent magnet DC motor of Fig 1. The state-space representation for permanent magnet DC motor provided in [44, 45] is given as

$$\begin{array}{l} \dot{i}=-\frac{a}{L}w-\frac{R}{L}\varsigma -\frac{c_{1}}{L}w\varsigma +\frac{1}{L}u_{sat},\\ \dot{w}=\frac{-F}{J}w+\frac{a}{J}\varsigma +\frac{c_{1}}{J}\varsigma^{2}-\frac{1}{J}T_{l}, \end{array}$$ (33)

where *R*, *L*, and *ς* represent the armature resistance, inductance, and current, respectively. *w*, *J*, and *F* indicate the motor rotational speed, inertia, and viscous frictional constant, respectively. *a* is the no load machine constant, *T*_*l*_ indicates the torque applied by an external load to the rotor, *c*_1_ represents a small negative number, and *u*_*sat*_ represents the saturated control signal. The parameters used in (33) are given in Table 1.

**Table 1:** Parameters of DC servo motor.

Physical Quantity	Symbol	Value	Units
Armature resistance	*R*	5.65	Ω
Inertia	*J*	1.88×10^−6^	Nms^2^
Armature inductance	*L*	2.90×10^−3^	H
Saturated input limit	*u*_*sat*_	12	V
Constant	*c*_1_	−0.01	Ω/rad
Friction constant	*F*	2.03×10^−5^	Nms
No load machine constant	*a*	2.39×10^−2^	V/rad

Considering ${\left[\begin{array}{cc} x_{1} & x_{2} \end{array}\right]}^{T}={\left[\begin{array}{cc} w & \varsigma \end{array}\right]}^{T}$ and by putting the values of the physical quantities of permanent magnet DC motor given in Table 1, the following nonlinear system matrices are obtain

$$\begin{array}{l} A=\left[\begin{array}{cc} -1.1 & 12724\\ -8.3 & -1956 \end{array}\right],B=\left[\begin{array}{c} 0\\ 355.43 \end{array}\right],\\ f(t,x)=\left[\begin{array}{c} -5453x_{2}^{2}\\ 3.54x_{1}x_{2} \end{array}\right],D=\left[\begin{array}{cc} 1 & 0 \end{array}\right],\\ C_{1}=C_{2}=\left[\begin{array}{cc} 1 & 0 \end{array}\right]. \end{array}$$ (34)

The following controller is designed using Matlab’s response optimization tool for the required tracking of motor speed without considering the saturation effects.


$$\begin{array}{l} {\dot{x}}_{cont}=e\\ u=0.053x_{cont}+0.00218e,\\ e=\bar{w}-y. \end{array}$$ (35)

Note that the controller (35) is same as (2) under *A*_*cont*_ = 0, *B*_*cont*_ = 1, *C*_*cont*_ = 0.053, and *D*_*cont*_ = 0.0021. The conditions of Theorem 2 have been solved to obtain the static AWC gain as *E*_*c*_ = 269.4990. The *L*_2_ gain from *w* to *z* is optimized by solving the routines of Theorem 2 for the optimization of *γ*. The best *L*_2_ gain *γ* = 1.00 is obtain from Theorem 2.

**Fig 2:**
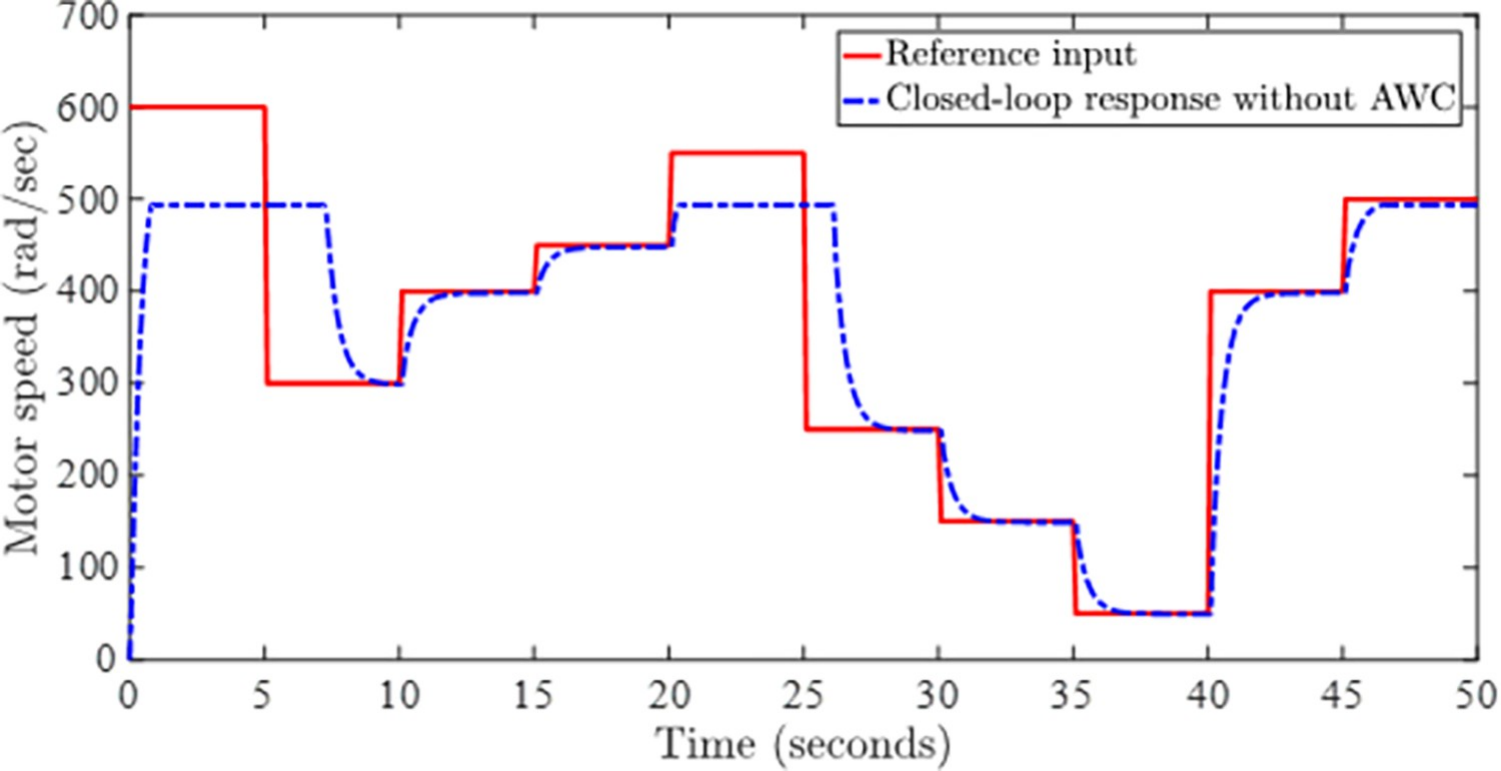
Closed-loop response without AWC.

**Fig 3:**
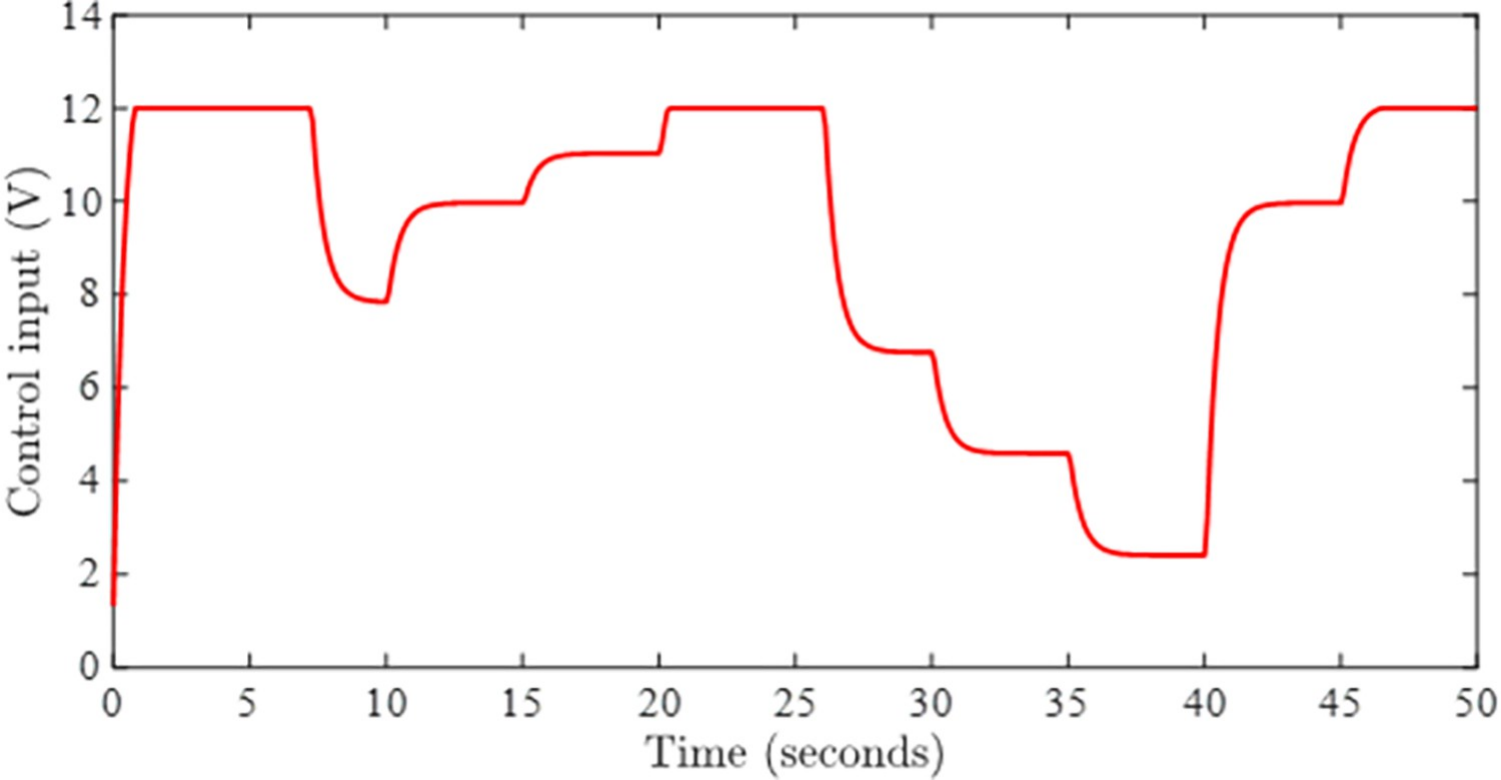
Control signal without AWC.

**Fig 4:**
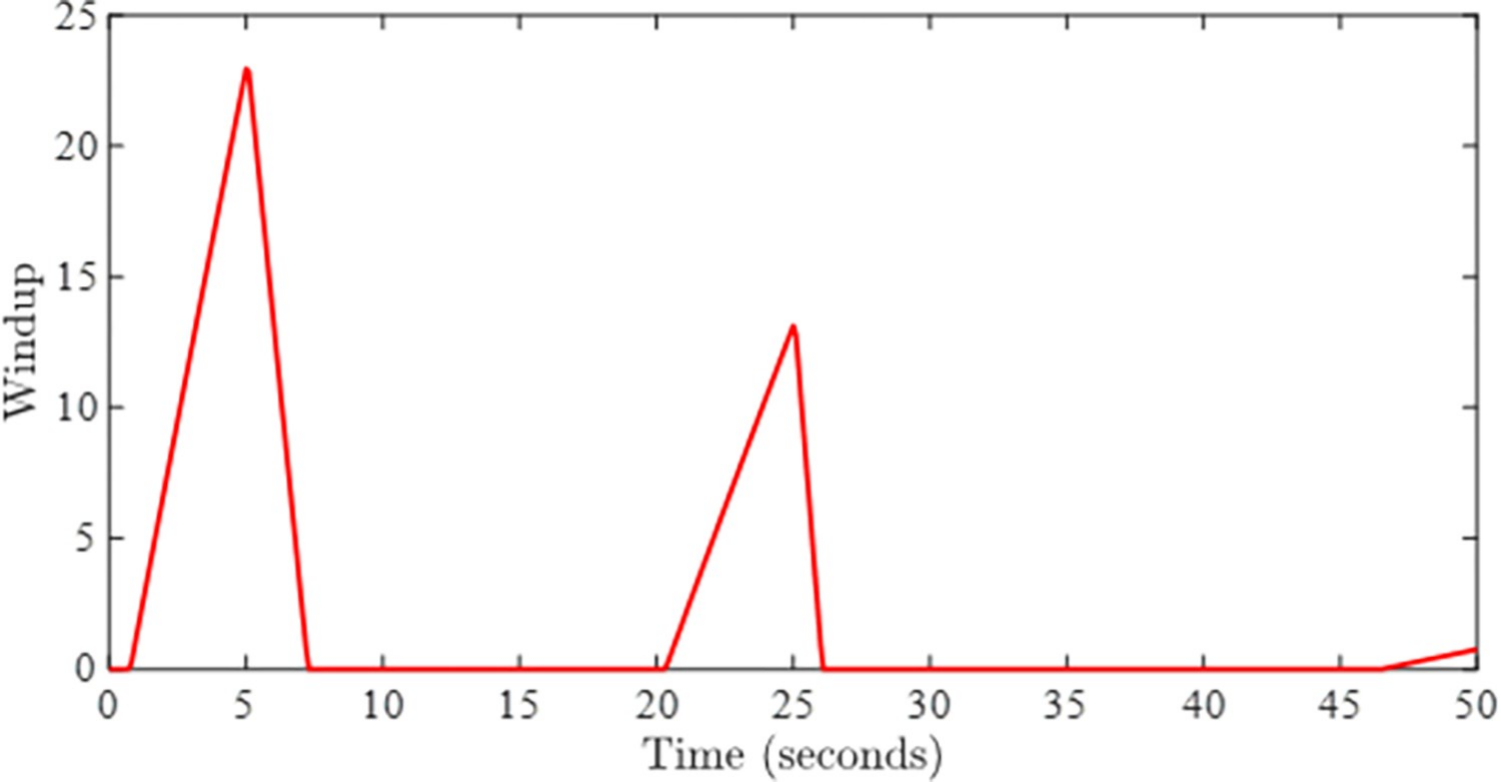
Windup effect without AWC.

Figs 2 and 3 depict the simulations that have been carried without incorporating an AWC in the nominal controller. Fig 2 shows the closed-loop output and input reference simultaneously in the presence of input and output delays. It can be observed from Fig 2 that the tracking of input reference is greatly affected when the input signal is beyond the saturation limit and windup effect due saturation can be seen in the form of lags. The windup effect can be observed in Fig 4 in the form of peaks whenever input signal goes beyond the saturation level. The magnitude of peaks goes up to 24 and it takes about 3 seconds to diminish if input exceeds the saturation limit by 100 rad/sec for 5 seconds.

**Fig 5:**
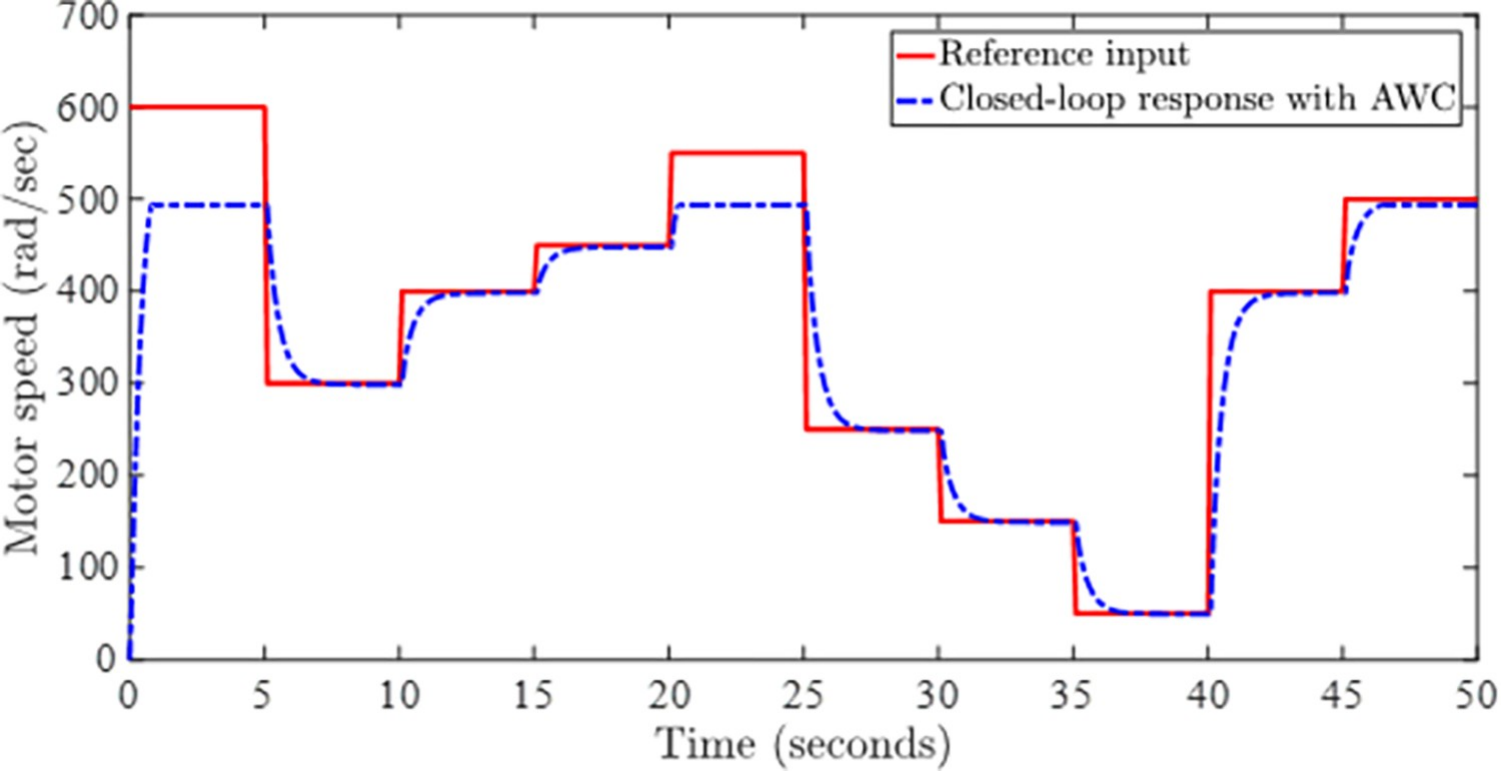
Closed-loop response with AWC.

**Fig 6:**
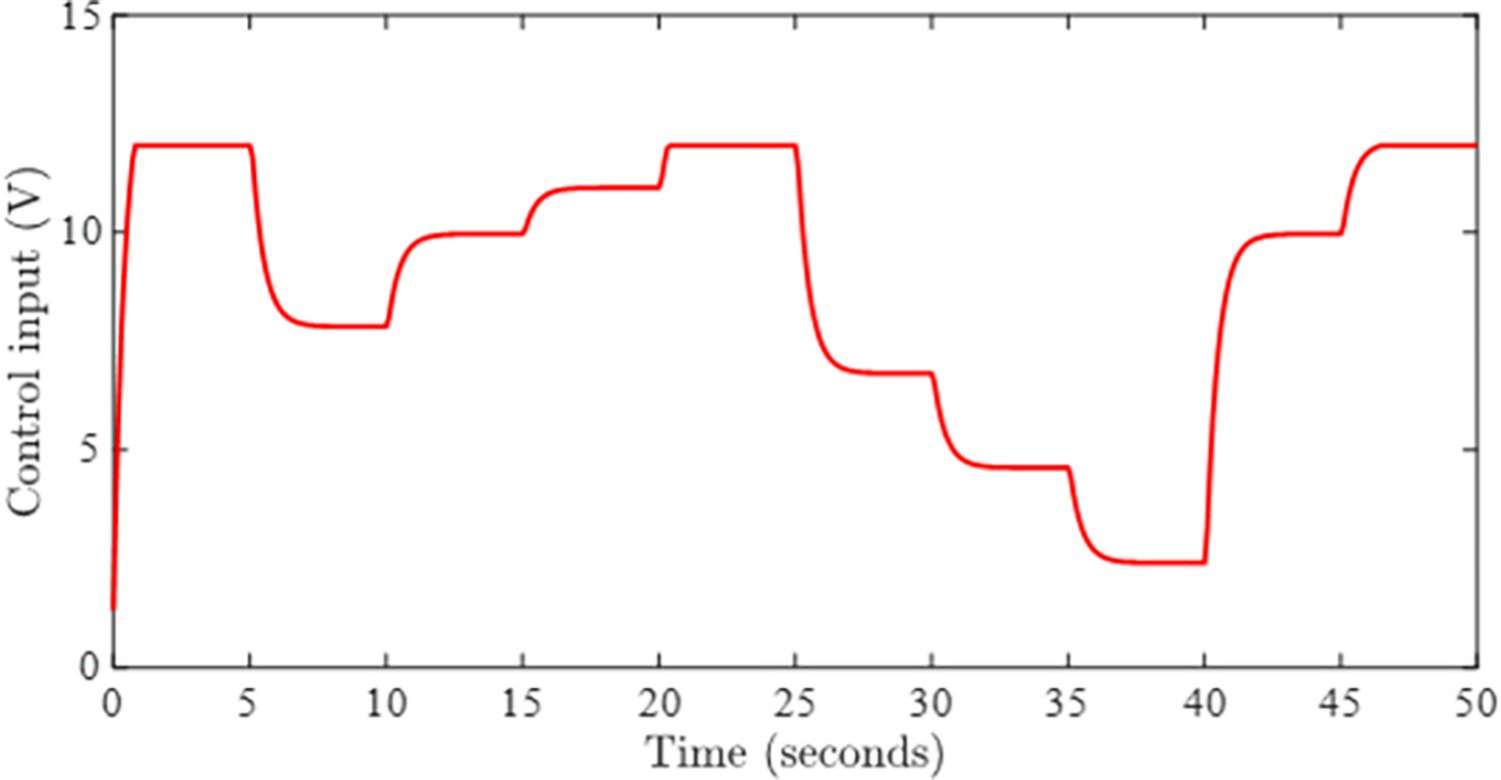
Control signal with AWC.

**Fig 7:**
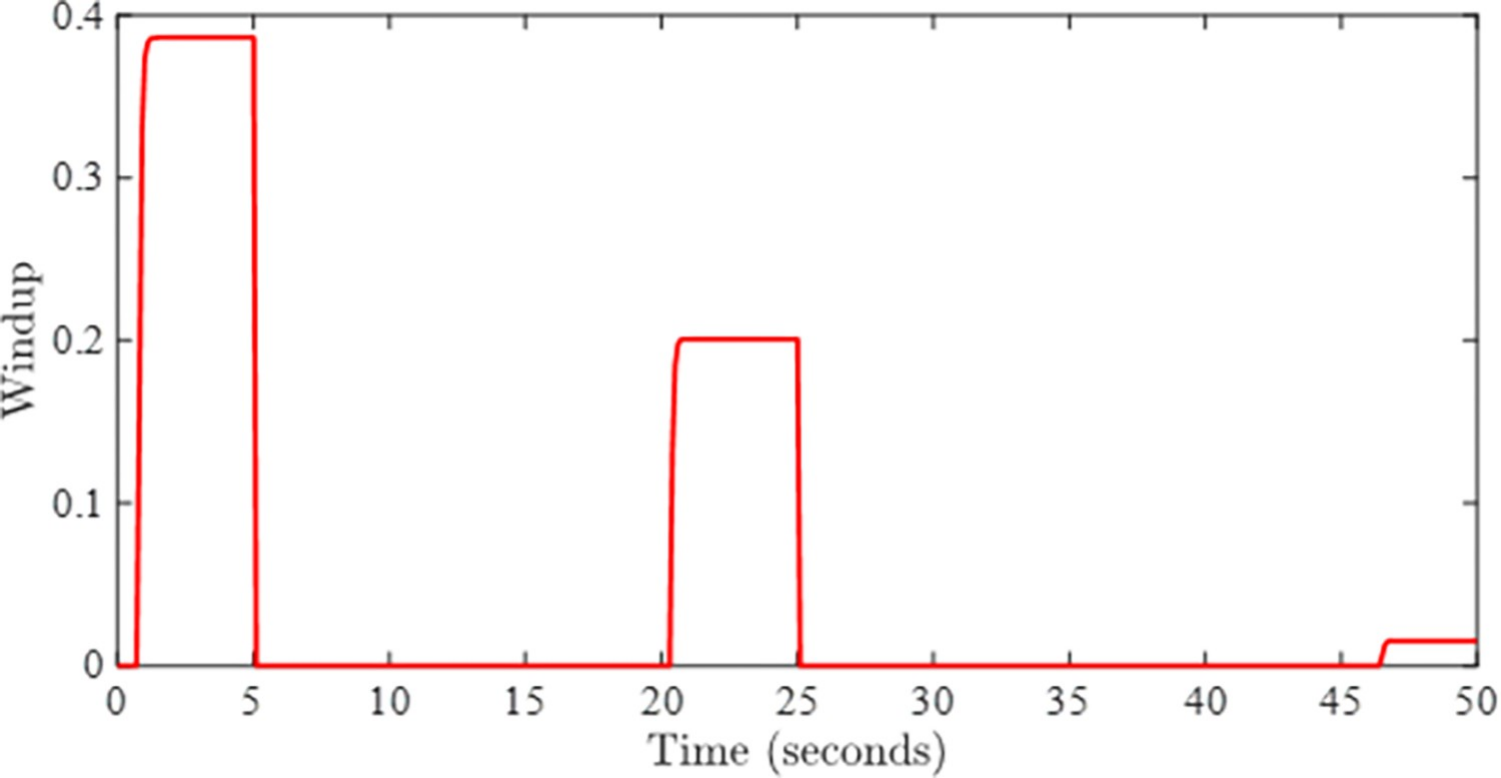
Windup effect with AWC.

Figs 5 and 6 show the tracking performance obtained by incorporating AWC gain into the controller under input saturation and time-varying delay. Fig 5 depicts the closed-loop response of the permanent magnet DC motor system by application of the proposed static AWC in the presence of input saturation. As it can be clearly observed from Fig 5 (see also from Fig 6 corresponding control signal) that windup effect (lags) in the tracking of motor speed is compensated by employing the proposed static AWC. Control signal is shown in Fig 6, which gets saturated after 12 V. Fig 7 depicts the windup effect with the proposed static AWC, which is quite negligible as compared to Fig 4.

**Fig 8:**
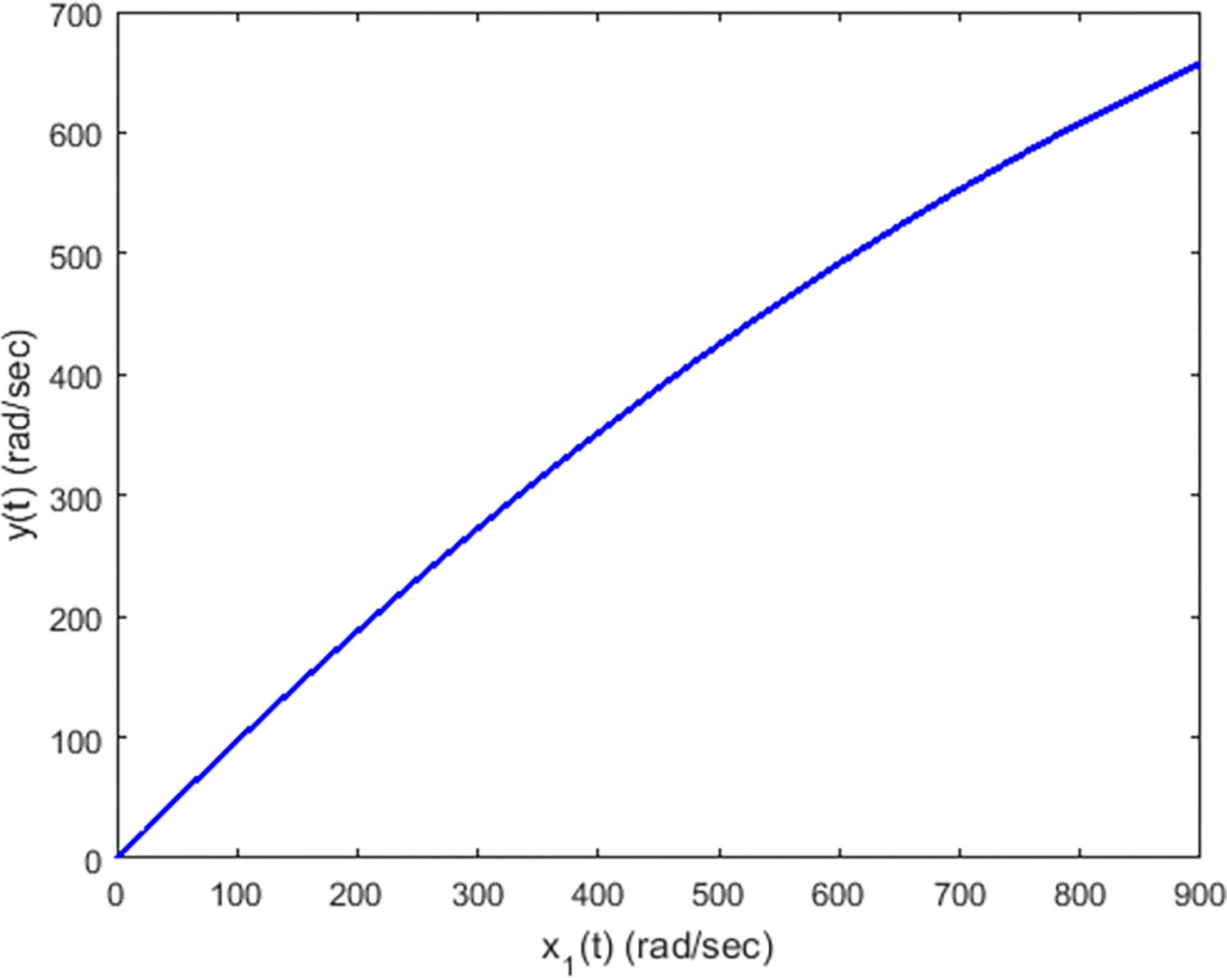
Sensor nonlinearity for DC motor speed control.

Now we consider another experiment for considering the sensor nonlinearity effects. The sensor nonlinearity can arise owing to a nonlinear behavior of a measurement device and cannot be avoided in practical systems. This sensor nonlinearity can cause saturation of sensor output, leading to a more perplexing practical situation. In the previous experiment, we have considered the output as linear such that *y*(*t*) = *x*_1_(*t*−*t*_2_). Now we assume that due to the sensor nonlinearity, the output is attained as nonlinear, given by $y(t)=x_{1}(t-\iota_{2})-0.0003x_{1}^{2}(t-\iota_{2})$. This type of nonlinearity can be typically modeled as a smooth sensor saturation. The graph between the outputs of a linear and a nonlinear sensor (that can be regarded as a plot between *x*_1_(*t*) and *y*(*t*)) has been shown in Fig 8. The output of the sensor changes in a nonlinear manner with respect to the actual (linear) angular speed.

**Fig 9:**
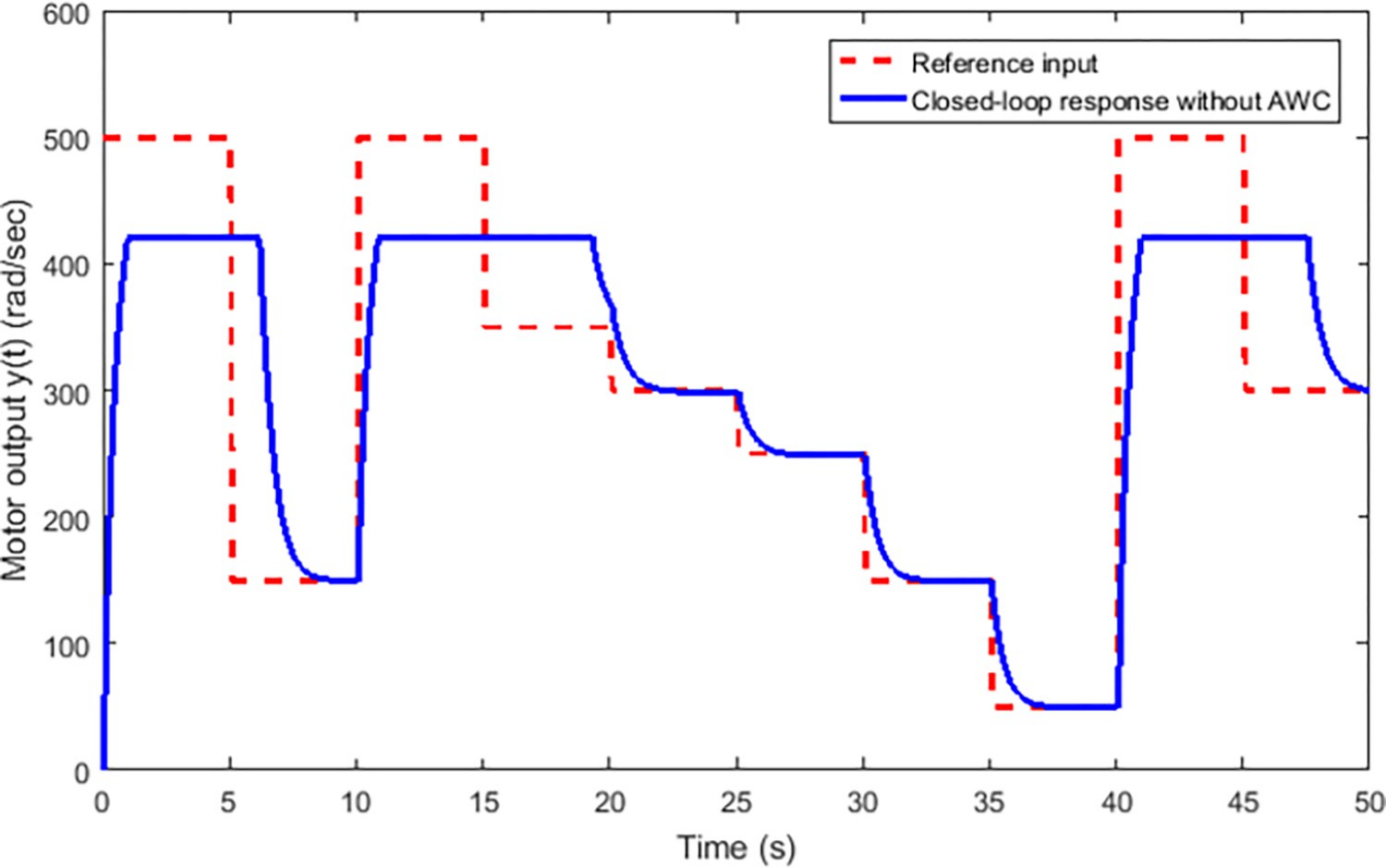
Response without AWC under sensor nonlinearity.

**Fig 10:**
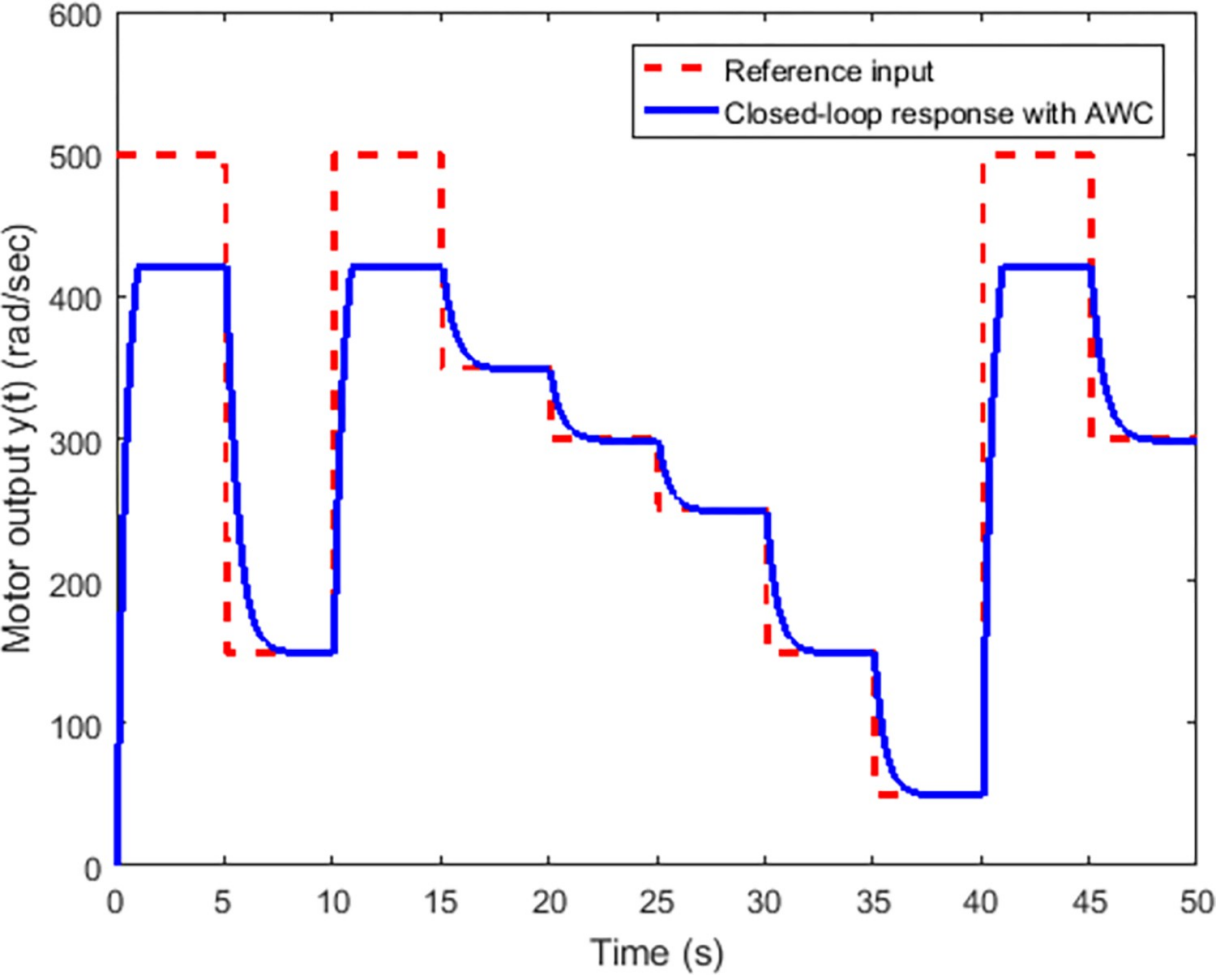
Response with the proposed AWC under sensor nonlinearity.

It is worth noting that the sensor nonlinearity can cause more sewer response of the closed-loop system in the presence of other complications, as the deflection of the sensor output is not same as for the physical quantity. This sensor nonlinearity effect has not been considered in the existing methods, such as [24, 37, 46–48]. Under the same controller settings, the response of the control system without AWC is demonstrated in Fig 9. Clearly, it can be observed that the windup effect is more swear because the sensor output has been saturated around 422 rad/sec (which is less than the previous case). In addition to this saturation, a lag in tracking of the reference signal has been observed owing to the so-called windup phenomenon. To overcome the lag issue, we have designed an AWC through the proposed method of Theorem 2. The gain of the AWC has been obtained as *E*_*c*_ = 245.92 for the parameter *γ* = 0.87. The outcome of the proposed AWC has been shown in Fig 10. It can be observed that the proposed method has removed the lag due to the windup effect in a more complex situation of the sensor nonlinearity. Hence, the proposed AWC can be applied to deal with the complicated situations for removal of windup effect in contrast to the existing methods.

## 5. Conclusions

In this paper, we have considered the design of a static AWC using a novel technique for the nonlinear locally Lipschitz systems with multiple range-dependent time-delays in input and output of the system. The nonlinearities in the system are considered to be locally Lipschitz in a bounded ellipsoidal region. An AWC is incorporated in the existing output feedback controller, designed to satisfy system performance without saturation. Techniques like *L*_2_ gain minimization, convex optimization, and Wirtinger inequality are utilized to design conditions for AWC gain. In contrast to the existing methods, the proposed approach is promising due to its application to the multiple delays (including additive time-varying delay) and owing to consideration of the locally Lipschitz nonlinearities, in addition to use of advanced control methods. The proposed design methodology is simple to design and it provides a better stability analysis owing to range-dependent time-delays. Theoretical results derived in this paper have been verified using numerical simulations for nonlinear DC motor. In future, the present study can be extended for dealing with large variations in time-delays. In addition, the scope of nonlinearity can also be extended for even broader class of locally one-sided Lipschitz nonlinear systems in the future studies.

## Supporting information

S1 Appendix (DOCX)
