# Supplementary material for: Static anti-windup compensator design for locally Lipschitz systems under input and output delays
Source: PLoS One. 2023 Apr 11;18(4):e0283734. doi: 10.1371/journal.pone.0283734 (PMC10089364; doi:10.1371/journal.pone.0283734)
Supplement: S1 Appendix — (DOCX) [file pone.0283734.s001.docx]

**Appendix A: The details of expressions in Theorem 1**

$$ϒ^{11}=\left[\begin{matrix} \Xi_{1} & 0 & 0 & 0 & 0 & PB_{11} & PB_{12} & PB_{13} & 0 & 0 & 0 & 0 & PF & PG_{1} & PG_{2} & \Xi_{11} & PW_{2} & \Xi_{9} & PY_{2} \\ * & -Q_{1} & 0 & 0 & 0 & 0 & 0 & 0 & 0 & 0 & 0 & 0 & 0 & 0 & 0 & 0 & 0 & 0 & 0 \\ * & * & -Q_{2} & 0 & 0 & 0 & 0 & 0 & 0 & 0 & 0 & 0 & 0 & 0 & 0 & 0 & 0 & 0 & 0 \\ * & * & * & -Q_{3} & 0 & 0 & 0 & 0 & 0 & 0 & 0 & 0 & 0 & 0 & 0 & 0 & 0 & 0 & 0 \\ * & * & * & * & -Q_{4} & 0 & 0 & 0 & 0 & 0 & 0 & 0 & 0 & 0 & 0 & 0 & 0 & 0 & 0 \\ * & * & * & * & * & \Xi_{2} & 0 & 0 & 0 & 0 & 0 & 0 & 0 & 0 & 0 & 0 & C_{s}^{T}D & 0 & NW \\ * & * & * & * & * & * & \Xi_{3} & 0 & 0 & 0 & 0 & 0 & 0 & 0 & 0 & 0 & 0 & D_{1}W & 0 \\ * & * & * & * & * & * & * & \Xi_{4} & 0 & 0 & 0 & 0 & 0 & 0 & 0 & 0 & 0 & 0 & D_{1}^{T}W \\ * & * & * & * & * & * & * & * & \Xi_{5} & 0 & 0 & 0 & 0 & 0 & 0 & 0 & 0 & 0 & 0 \\ * & * & * & * & * & * & * & * & * & \Xi_{6} & 0 & 0 & 0 & 0 & 0 & 0 & 0 & 0 & 0 \\ * & * & * & * & * & * & * & * & * & * & \Xi_{7} & 0 & 0 & 0 & 0 & 0 & 0 & 0 & 0 \\ * & * & * & * & * & * & * & * & * & * & * & \Xi_{8} & 0 & 0 & 0 & 0 & 0 & 0 & 0 \\ * & * & * & * & * & * & * & * & * & * & * & * & -I & 0 & 0 & 0 & 0 & 0 & 0 \\ * & * & * & * & * & * & * & * & * & * & * & * & * & -I & 0 & 0 & 0 & D_{cont}^{T}W & 0 \\ * & * & * & * & * & * & * & * & * & * & * & * & * & * & -I & 0 & 0 & 0 & D_{cont}^{T}W \\ * & * & * & * & * & * & * & * & * & * & * & * & * & * & * & \Xi_{10} & 0 & D_{cont}^{T}W & 0 \\ * & * & * & * & * & * & * & * & * & * & * & * & * & * & * & * & \Xi_{10} & 0 & D_{cont}^{T}W \\ * & * & * & * & * & * & * & * & * & * & * & * & * & * & * & * & * & -2W & 0 \\ * & * & * & * & * & * & * & * & * & * & * & * & * & * & * & * & * & * & -2W \end{matrix}\right],$$

$$ϒ^{12}=\left[O_{19\times12}\right],\text{ }ϒ^{22}=\left[O_{12\times12}\right],$$

$T_{1}=\left[\begin{matrix} G_{3}^{T} & G_{4}^{T} & G_{5}^{T} & G_{6}^{T} \end{matrix}\right]^{T}$,$T_{2}=\left[\begin{matrix} G_{7}^{T} & G_{8}^{T} & G_{5}^{T} & G_{6}^{T} \end{matrix}\right]^{T}$,

$T_{3}=\left[\begin{matrix} G_{9}^{T} & G_{10}^{T} & G_{11}^{T} & G_{12}^{T} \end{matrix}\right]^{T}$, $T_{4}=\left[\begin{matrix} G_{13}^{T} & G_{14}^{T} & G_{11}^{T} & G_{12}^{T} \end{matrix}\right]^{T}$, $T_{5}=\left[\begin{matrix} G_{15}^{T} & G_{16}^{T} & G_{17}^{T} & G_{18}^{T} \end{matrix}\right]^{T}$ $T_{6}=\left[\begin{matrix} G_{19}^{T} & G_{20}^{T} & G_{17}^{T} & G_{18}^{T} \end{matrix}\right]^{T}$, $T_{7}=\left[\begin{matrix} G_{21}^{T} & G_{22}^{T} & G_{23}^{T} & G_{24}^{T} \end{matrix}\right]^{T}$, $T_{8}=\left[\begin{matrix} G_{25}^{T} & G_{26}^{T} & G_{23}^{T} & G_{24}^{T} \end{matrix}\right]^{T}$,

$$G_{3}=\left[\begin{matrix} I & O_{1\times4} & -I & O_{1\times25} \end{matrix}\right],$$

$$G_{4}=\left[\begin{matrix} I & O_{1\times4} & I & O_{1\times13} & -2I & O_{1\times11} \end{matrix}\right],$$

$$G_{5}=\left[\begin{matrix} O_{1\times2} & -I & O_{1\times2} & I & O_{1\times25} \end{matrix}\right],$$

$$G_{6}=\left[\begin{matrix} O_{1\times2} & I & O_{1\times2} & I & O_{1\times14} & -2I & O_{1\times10} \end{matrix}\right],$$

$$G_{7}=\left[\begin{matrix} O_{1\times1} & I & O_{1\times3} & -I & O_{1\times25} \end{matrix}\right],$$

$$G_{8}=\left[\begin{matrix} O_{1\times1} & I & O_{1\times3} & I & O_{1\times15} & 2I & O_{1\times9} \end{matrix}\right],$$

$$G_{9}=\left[\begin{matrix} I & O_{1\times5} & -I & O_{1\times24} \end{matrix}\right],$$

$$G_{10}=\left[\begin{matrix} I & O_{1\times5} & I & O_{1\times15} & -2I & O_{1\times8} \end{matrix}\right],$$

$$G_{11}=\left[\begin{matrix} O_{1\times4} & -I & O_{1\times1} & I & O_{1\times24} \end{matrix}\right],$$

$$G_{12}=\left[\begin{matrix} O_{1\times4} & I & O_{1\times1} & I & O_{1\times16} & -2I & O_{1\times7} \end{matrix}\right],$$

$$G_{13}=\left[\begin{matrix} O_{1\times3} & I & O_{1\times2} & -I & O_{1\times24} \end{matrix}\right],$$

$$G_{14}=\left[\begin{matrix} O_{1\times3} & I & O_{1\times2} & I & O_{1\times17} & -2I & O_{1\times6} \end{matrix}\right],$$

$$G_{15}=\left[\begin{matrix} O_{1\times5} & I & O_{1\times1} & -I & O_{1\times23} \end{matrix}\right],$$

$$G_{16}=\left[\begin{matrix} O_{1\times5} & I & O_{1\times1} & I & O_{1\times20} & -2I & O_{1\times2} \end{matrix}\right],$$

$$G_{17}=\left[\begin{matrix} O_{1\times7} & I & O_{1\times1} & -I & O_{1\times21} \end{matrix}\right],$$

$$G_{18}=\left[\begin{matrix} O_{1\times71} & I & O_{1\times1} & I & O_{1\times19} & -2I & O_{1\times1} \end{matrix}\right],$$

$$G_{19}=\left[\begin{matrix} O_{1\times3} & I & O_{1\times3} & -I & O_{1\times23} \end{matrix}\right],$$

$$G_{20}=\left[\begin{matrix} O_{1\times3} & I & O_{1\times3} & I & O_{1\times22} & -2I \end{matrix}\right],$$

$$G_{21}=\left[\begin{matrix} O_{1\times6} & I & -I & O_{1\times23} \end{matrix}\right],$$

$$G_{22}=\left[\begin{matrix} O_{1\times6} & I & I & O_{1\times17} & -2I & O_{1\times5} \end{matrix}\right],$$

$$G_{23}=\left[\begin{matrix} O_{1\times7} & I & O_{1\times3} & -I & O_{1\times19} \end{matrix}\right],$$

$$G_{24}=\left[\begin{matrix} O_{1\times7} & I & O_{1\times3} & I & O_{1\times15} & -2I & O_{1\times3} \end{matrix}\right],$$

$$G_{25}=\left[\begin{matrix} O_{1\times7} & -I & O_{1\times2} & I & O_{1\times20} \end{matrix}\right],$$

$$G_{26}=\left[\begin{matrix} O_{1\times7} & I & O_{1\times2} & I & O_{1\times15} & -2I & O_{1\times4} \end{matrix}\right],$$

$$\begin{matrix} \Xi_{1}=PA_{11}+A_{11}^{T}P+\sum_{j=1}^{4} Q_{j}+Q_{7}+Q_{8}+L_{1}^{T}L_{1}+C_{s}^{T}C_{s},\text{ } \\ \Xi_{5}=-(1-u_{1})Q_{11},\text{ }\Xi_{6}=-(1-u_{1})Q_{12}, \\ \Xi_{2}=-(1-u_{1})Q_{7}+Q_{1}+Q_{11}+Q_{12}+L_{2}^{T}L_{2}+C_{s}^{T}C_{s},\text{ } \\ \Xi_{6}=-(1-u_{1})Q_{12},\text{ }\Xi_{7}=-(1-u_{2})Q_{13}, \\ \Xi_{3}=-(1-u_{2})Q_{8}+Q_{10}+Q_{13}+Q_{14}+L_{2}^{T}L_{2},\text{ } \\ \Xi_{8}=-(1-u_{2})Q_{14},\text{ }\Xi_{9}=PY_{1}E_{c}+N^{T}W, \\ \Xi_{4}=-(1-u_{1}-u_{2})Q_{9}-(1-u_{1}-u_{2})Q_{10},\text{ } \\ \Xi_{10}=-\rho I+D^{T}D,\text{ }\Xi_{11}=PW_{1}+C_{s}^{T}D, \\ h_{21}=h_{2}-h_{1},\text{ }h_{34}=h_{4}-h_{3},\text{ }\iota_{3}=\iota_{1}+\iota_{2}. \end{matrix}$$

**Appendix B: The details of expressions in Theorem 2**

$$\Delta^{11}=\left[\begin{matrix} \tilde{\Xi}_{1} & 0 & 0 & 0 & 0 & B_{11}X & B_{12}X & B_{13}X & 0 & 0 & 0 & 0 & F & G_{1} & G_{2} & \tilde{\Xi}_{11} & W_{2} & \tilde{\Xi}_{9} & Y_{2}U \\ * & -\tilde{Q}_{1} & 0 & 0 & 0 & 0 & 0 & 0 & 0 & 0 & 0 & 0 & 0 & 0 & 0 & 0 & 0 & 0 & 0 \\ * & * & -\tilde{Q}_{2} & 0 & 0 & 0 & 0 & 0 & 0 & 0 & 0 & 0 & 0 & 0 & 0 & 0 & 0 & 0 & 0 \\ * & * & * & -\tilde{Q}_{3} & 0 & 0 & 0 & 0 & 0 & 0 & 0 & 0 & 0 & 0 & 0 & 0 & 0 & 0 & 0 \\ * & * & * & * & -\tilde{Q}_{4} & 0 & 0 & 0 & 0 & 0 & 0 & 0 & 0 & 0 & 0 & 0 & 0 & 0 & 0 \\ * & * & * & * & * & \tilde{\Xi}_{2} & 0 & 0 & 0 & 0 & 0 & 0 & 0 & 0 & 0 & 0 & 0 & 0 & \bar{N} \\ * & * & * & * & * & * & \tilde{\Xi}_{3} & 0 & 0 & 0 & 0 & 0 & 0 & 0 & 0 & 0 & 0 & XD_{1}^{T} & 0 \\ * & * & * & * & * & * & * & \tilde{\Xi}_{4} & 0 & 0 & 0 & 0 & 0 & 0 & 0 & 0 & 0 & 0 & XD_{1}^{T} \\ * & * & * & * & * & * & * & * & \tilde{\Xi}_{5} & 0 & 0 & 0 & 0 & 0 & 0 & 0 & 0 & 0 & 0 \\ * & * & * & * & * & * & * & * & * & \tilde{\Xi}_{6} & 0 & 0 & 0 & 0 & 0 & 0 & 0 & 0 & 0 \\ * & * & * & * & * & * & * & * & * & * & \tilde{\Xi}_{7} & 0 & 0 & 0 & 0 & 0 & 0 & 0 & 0 \\ * & * & * & * & * & * & * & * & * & * & * & \tilde{\Xi}_{8} & 0 & 0 & 0 & 0 & 0 & 0 & 0 \\ * & * & * & * & * & * & * & * & * & * & * & * & -I & 0 & 0 & 0 & 0 & 0 & 0 \\ * & * & * & * & * & * & * & * & * & * & * & * & * & -I & 0 & 0 & 0 & D_{cont}^{T} & 0 \\ * & * & * & * & * & * & * & * & * & * & * & * & * & * & -I & 0 & 0 & 0 & D_{cont}^{T} \\ * & * & * & * & * & * & * & * & * & * & * & * & * & * & * & \tilde{\Xi}_{10} & 0 & D_{cont}^{T} & 0 \\ * & * & * & * & * & * & * & * & * & * & * & * & * & * & * & * & \tilde{\Xi}_{10} & 0 & D_{cont}^{T} \\ * & * & * & * & * & * & * & * & * & * & * & * & * & * & * & * & * & -2U & 0 \\ * & * & * & * & * & * & * & * & * & * & * & * & * & * & * & * & * & * & -2U \end{matrix}\right],$$

$$\Delta^{12}=\left[\begin{matrix} 0 & 0 & 0 & 0 & 0 & 0 & 0 & 0 & 0 & 0 & 0 & 0 & XA_{11}^{T} & XA_{11}^{T} & XA_{11}^{T} & XA_{11}^{T} & XA_{11}^{T} & XA_{11}^{T} & XA_{11}^{T} & XA_{11}^{T} \\ 0 & 0 & 0 & 0 & 0 & 0 & 0 & 0 & 0 & 0 & 0 & 0 & 0 & 0 & 0 & 0 & 0 & 0 & 0 & 0 \\ 0 & 0 & 0 & 0 & 0 & 0 & 0 & 0 & 0 & 0 & 0 & 0 & 0 & 0 & 0 & 0 & 0 & 0 & 0 & 0 \\ 0 & 0 & 0 & 0 & 0 & 0 & 0 & 0 & 0 & 0 & 0 & 0 & 0 & 0 & 0 & 0 & 0 & 0 & 0 & 0 \\ 0 & 0 & 0 & 0 & 0 & 0 & 0 & 0 & 0 & 0 & 0 & 0 & 0 & 0 & 0 & 0 & 0 & 0 & 0 & 0 \\ 0 & 0 & 0 & 0 & 0 & 0 & 0 & 0 & 0 & 0 & 0 & 0 & XB_{11}^{T} & XB_{11}^{T} & XB_{11}^{T} & XB_{11}^{T} & XB_{11}^{T} & XB_{11}^{T} & XB_{11}^{T} & XB_{11}^{T} \\ 0 & 0 & 0 & 0 & 0 & 0 & 0 & 0 & 0 & 0 & 0 & 0 & XB_{12}^{T} & XB_{12}^{T} & XB_{12}^{T} & XB_{12}^{T} & XB_{12}^{T} & XB_{12}^{T} & XB_{12}^{T} & XB_{12}^{T} \\ 0 & 0 & 0 & 0 & 0 & 0 & 0 & 0 & 0 & 0 & 0 & 0 & XB_{13}^{T} & XB_{13}^{T} & XB_{13}^{T} & XB_{13}^{T} & XB_{13}^{T} & XB_{13}^{T} & XB_{13}^{T} & XB_{13}^{T} \\ 0 & 0 & 0 & 0 & 0 & 0 & 0 & 0 & 0 & 0 & 0 & 0 & 0 & 0 & 0 & 0 & 0 & 0 & 0 & 0 \\ 0 & 0 & 0 & 0 & 0 & 0 & 0 & 0 & 0 & 0 & 0 & 0 & 0 & 0 & 0 & 0 & 0 & 0 & 0 & 0 \\ 0 & 0 & 0 & 0 & 0 & 0 & 0 & 0 & 0 & 0 & 0 & 0 & 0 & 0 & 0 & 0 & 0 & 0 & 0 & 0 \\ 0 & 0 & 0 & 0 & 0 & 0 & 0 & 0 & 0 & 0 & 0 & 0 & 0 & 0 & 0 & 0 & 0 & 0 & 0 & 0 \\ 0 & 0 & 0 & 0 & 0 & 0 & 0 & 0 & 0 & 0 & 0 & 0 & F^{T} & F^{T} & F^{T} & F^{T} & F^{T} & F^{T} & F^{T} & F^{T} \\ 0 & 0 & 0 & 0 & 0 & 0 & 0 & 0 & 0 & 0 & 0 & 0 & G_{1}^{T} & G_{1}^{T} & G_{1}^{T} & G_{1}^{T} & G_{1}^{T} & G_{1}^{T} & G_{1}^{T} & G_{1}^{T} \\ 0 & 0 & 0 & 0 & 0 & 0 & 0 & 0 & 0 & 0 & 0 & 0 & G_{2}^{T} & G_{2}^{T} & G_{2}^{T} & G_{2}^{T} & G_{2}^{T} & G_{2}^{T} & G_{2}^{T} & G_{2}^{T} \\ 0 & 0 & 0 & 0 & 0 & 0 & 0 & 0 & 0 & 0 & 0 & 0 & W_{1}^{T} & W_{1}^{T} & W_{1}^{T} & W_{1}^{T} & W_{1}^{T} & W_{1}^{T} & W_{1}^{T} & W_{1}^{T} \\ 0 & 0 & 0 & 0 & 0 & 0 & 0 & 0 & 0 & 0 & 0 & 0 & W_{2}^{T} & W_{2}^{T} & W_{2}^{T} & W_{2}^{T} & W_{2}^{T} & W_{2}^{T} & W_{2}^{T} & W_{2}^{T} \\ 0 & 0 & 0 & 0 & 0 & 0 & 0 & 0 & 0 & 0 & 0 & 0 & V^{T}Y_{1}^{T} & V^{T}Y_{1}^{T} & V^{T}Y_{1}^{T} & V^{T}Y_{1}^{T} & V^{T}Y_{1}^{T} & V^{T}Y_{1}^{T} & V^{T}Y_{1}^{T} & V^{T}Y_{1}^{T} \\ 0 & 0 & 0 & 0 & 0 & 0 & 0 & 0 & 0 & 0 & 0 & 0 & UY_{2}^{T} & UY_{2}^{T} & UY_{2}^{T} & UY_{2}^{T} & UY_{2}^{T} & UY_{2}^{T} & UY_{2}^{T} & UY_{2}^{T} \end{matrix}\right],$$

$$\Delta^{22}=\left[\begin{matrix} 0 & 0 & 0 & 0 & 0 & 0 & 0 & 0 & 0 & 0 & 0 & 0 & 0 & 0 & 0 & 0 & 0 & 0 & 0 & 0 \\ * & 0 & 0 & 0 & 0 & 0 & 0 & 0 & 0 & 0 & 0 & 0 & 0 & 0 & 0 & 0 & 0 & 0 & 0 & 0 \\ * & * & 0 & 0 & 0 & 0 & 0 & 0 & 0 & 0 & 0 & 0 & 0 & 0 & 0 & 0 & 0 & 0 & 0 & 0 \\ * & * & * & 0 & 0 & 0 & 0 & 0 & 0 & 0 & 0 & 0 & 0 & 0 & 0 & 0 & 0 & 0 & 0 & 0 \\ * & * & * & * & 0 & 0 & 0 & 0 & 0 & 0 & 0 & 0 & 0 & 0 & 0 & 0 & 0 & 0 & 0 & 0 \\ * & * & * & * & * & 0 & 0 & 0 & 0 & 0 & 0 & 0 & 0 & 0 & 0 & 0 & 0 & 0 & 0 & 0 \\ * & * & * & * & * & * & 0 & 0 & 0 & 0 & 0 & 0 & 0 & 0 & 0 & 0 & 0 & 0 & 0 & 0 \\ * & * & * & * & * & * & * & 0 & 0 & 0 & 0 & 0 & 0 & 0 & 0 & 0 & 0 & 0 & 0 & 0 \\ * & * & * & * & * & * & * & * & 0 & 0 & 0 & 0 & 0 & 0 & 0 & 0 & 0 & 0 & 0 & 0 \\ * & * & * & * & * & * & * & * & * & 0 & 0 & 0 & 0 & 0 & 0 & 0 & 0 & 0 & 0 & 0 \\ * & * & * & * & * & * & * & * & * & * & 0 & 0 & 0 & 0 & 0 & 0 & 0 & 0 & 0 & 0 \\ * & * & * & * & * & * & * & * & * & * & * & 0 & 0 & 0 & 0 & 0 & 0 & 0 & 0 & 0 \\ * & * & * & * & * & * & * & * & * & * & * & * & \Xi_{12} & 0 & 0 & 0 & 0 & 0 & 0 & 0 \\ * & * & * & * & * & * & * & * & * & * & * & * & * & \Xi_{13} & 0 & 0 & 0 & 0 & 0 & 0 \\ * & * & * & * & * & * & * & * & * & * & * & * & * & * & \Xi_{14} & 0 & 0 & 0 & 0 & 0 \\ * & * & * & * & * & * & * & * & * & * & * & * & * & * & * & \Xi_{15} & 0 & 0 & 0 & 0 \\ * & * & * & * & * & * & * & * & * & * & * & * & * & * & * & * & \Xi_{16} & 0 & 0 & 0 \\ * & * & * & * & * & * & * & * & * & * & * & * & * & * & * & * & * & \Xi_{17} & 0 & 0 \\ * & * & * & * & * & * & * & * & * & * & * & * & * & * & * & * & * & * & \Xi_{18} & 0 \\ * & * & * & * & * & * & * & * & * & * & * & * & * & * & * & * & * & * & * & \Xi_{19} \end{matrix}\right],\Delta^{13}=\left[\begin{matrix} XL_{1}^{T} & 0 & 0 & XC_{s}^{T} & 0 \\ 0 & 0 & 0 & 0 & 0 \\ 0 & 0 & 0 & 0 & 0 \\ 0 & 0 & 0 & 0 & 0 \\ 0 & 0 & 0 & 0 & 0 \\ 0 & XL_{2}^{T} & 0 & 0 & 0 \\ 0 & 0 & XL_{2}^{T} & 0 & 0 \\ 0 & 0 & 0 & 0 & 0 \\ 0 & 0 & 0 & 0 & 0 \\ 0 & 0 & 0 & 0 & 0 \\ 0 & 0 & 0 & 0 & 0 \\ 0 & 0 & 0 & 0 & 0 \\ 0 & 0 & 0 & 0 & 0 \\ 0 & 0 & 0 & 0 & 0 \\ 0 & 0 & 0 & 0 & 0 \\ 0 & 0 & 0 & D^{T} & 0 \\ 0 & 0 & 0 & 0 & D^{T} \\ 0 & 0 & 0 & 0 & 0 \\ 0 & 0 & 0 & 0 & 0 \end{matrix}\right],$$

$$\Delta^{33}=diag\{-I,-I,-I,-I,-I\},$$

$$\Delta^{23}=O_{20\times5},$$

$$\begin{matrix} \tilde{\Xi}_{1}=A_{11}X+XA_{11}^{T}+\tilde{Q}_{1}+\tilde{Q}_{2}+\tilde{Q}_{4}+\tilde{Q}_{3}+\tilde{Q}_{7}+\tilde{Q}_{8},\text{ } \\ \tilde{\Xi}_{2}=-(1-u_{1})\tilde{Q}_{7}+\tilde{Q}_{1}+\tilde{Q}_{11}+\tilde{Q}_{12},\text{ }\tilde{\Xi}_{6}=-(1-u_{1})\tilde{Q}_{12}, \\ \tilde{\Xi}_{3}=-(1-u_{2})\tilde{Q}_{8}+\tilde{Q}_{10}+\tilde{Q}_{13}+\tilde{Q}_{14},\text{ }\tilde{\Xi}_{7}=-(1-u_{2})\tilde{Q}_{13}, \\ \tilde{\Xi}_{4}=-(1-u_{1}-u_{2})\tilde{Q}_{9}-(1-u_{1}-u_{2})\tilde{Q}_{10},\text{ }\tilde{\Xi}_{8}=-(1-u_{2})\tilde{Q}_{14}, \end{matrix}$$

$$\begin{matrix} \tilde{\Xi}_{5}=-(1-u_{1})\tilde{Q}_{11},\text{ }\Xi_{12}=-h_{2}^{-1}X\tilde{Z}^{-1}_{1}X,\text{ }\tilde{\Xi}_{11}=W_{1}, \\ \tilde{\Xi}_{9}=Y_{1}V+\bar{N},\text{ }\Xi_{13}=-h_{2}^{-1}X\tilde{Z}^{-1}_{7}X,\text{ }\Xi_{15}=-h_{12}^{-1}X\tilde{Z}^{-1}_{8}X, \\ \tilde{\Xi}_{10}=-\rho I,\text{ }\Xi_{16}=-h_{4}^{-1}X\tilde{Z}^{-1}_{3}X,\text{ }\Xi_{17}=-h_{4}^{-1}X\tilde{Z}^{-1}_{5}X, \\ \Xi_{18}=-h_{34}^{-1}X\tilde{Z}^{-1}_{4}X,\text{ }\Xi_{14}=-h_{12}^{-1}X\tilde{Z}^{-1}_{2}X,\Xi_{19}=-h_{34}^{-1}X\tilde{Z}^{-1}_{6}X, \end{matrix}$$

$G_{l}^{'}=\left[\begin{matrix} G_{l}^{T} & O_{1\times13} \end{matrix}\right]^{T},$ $G_{ij}^{'}=\left[\begin{matrix} G_{ij}^{T} & O_{1\times13} \end{matrix}\right]^{T}$.
